# Supplementary material for: Acute kidney injury in the UK: a replication cohort study of the variation across three regional populations
Source: BMJ Open. 2018 Jun 30;8(6):e019435. doi: 10.1136/bmjopen-2017-019435 (PMC6042563; doi:10.1136/bmjopen-2017-019435)

Supplemental table 1 – AKI definition and phenotype criteria for this study

AKI Criteria	AKI definition
Criterion 1	Serum creatinine ≥1.5 times higher than the median of all creatinine values 8-90 days ago, or 91-365 days ago if no tests between 8-90 days
Criterion 2	Serum creatinine ≥1.5 times higher than the lowest creatinine within 7 days
Criterion 3	Serum creatinine >26 μmol/L higher than the lowest creatinine within 48 hours
**AKI severity**	**Staging definition (based on peak creatinine within 90 days of diagnosis)**
Stage 1	Rise in creatinine of >26 μmol/L; or index/baseline ratio ≥1.5 and <2
Stage 2	Index/baseline ratio ≥2 and <3
Stage 3	Index/baseline ratio ≥3; or ≥1.5 and index creatinine >354 μmol/L
**Prior AKI episodes**	**Prior AKI definition**
No prior AKI	AKI episode not preceded by any previous AKI episodes in the prior 3 years
Prior AKI	AKI episode preceded by at least one previous AKI episode in the prior 3 years
Recent prior AKI	AKI episode preceded by at least one previous AKI episode in the prior 1 year
**90 day AKI recovery**	**Recovery definition**
Recovery	Last creatinine within 90 days of AKI <1.2 times higher than the baseline creatinine at diagnosis
Non-recovery	Last creatinine within 90 days of AKI ≥1.2 times higher than the baseline creatinine at diagnosis, or still receiving acute RRT
"Untested"	No repeat blood tests taken within 90 days of AKI diagnosis

Abbreviations: AKI, acute kidney injury; RRT, renal replacement therapy

Supplementary figure 1 – Map of the UK populations in this analysis

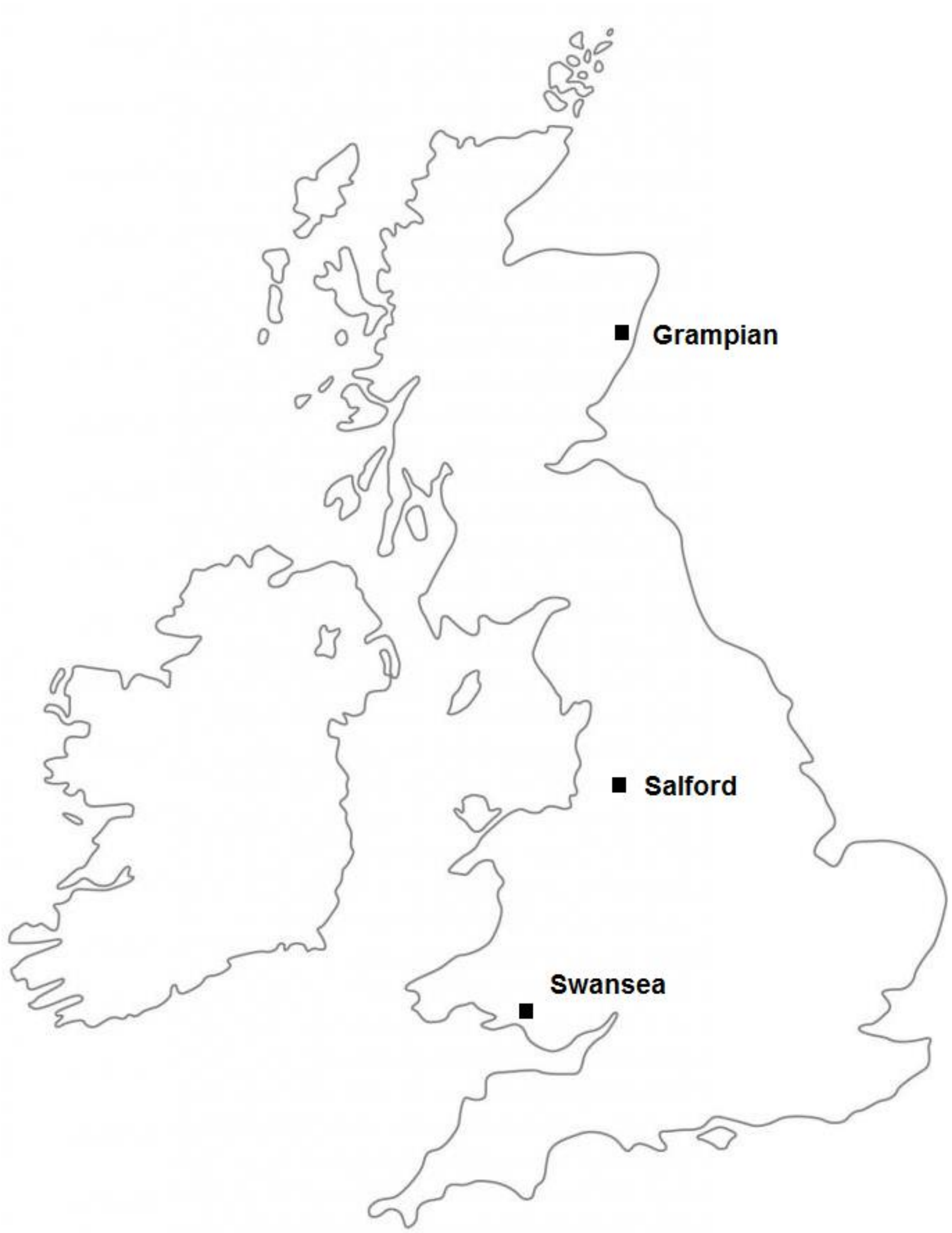

Supplemental figure 2 – Hypothetical patient illustrating look-back (for baseline) and look-forward (for AKI episode phenotyping) time periods from the start of an AKI episode

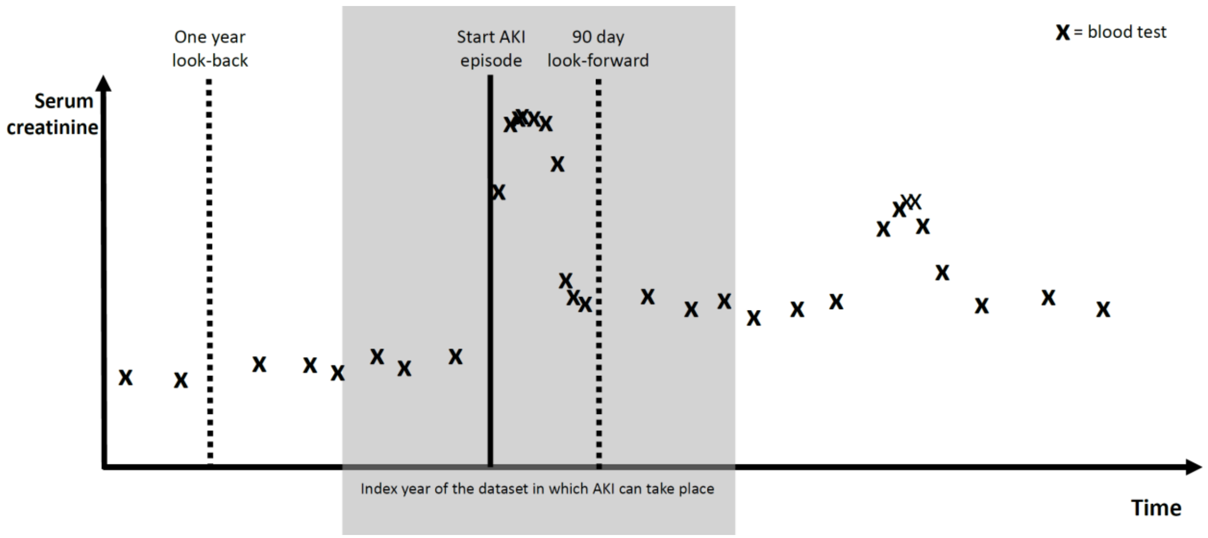

Supplement: Supplementary file 1 [file bmjopen-2017-019435supp001.pdf]
