# Supplementary material for: Acute kidney injury in the UK: a replication cohort study of the variation across three regional populations
Source: BMJ Open. 2018 Jun 30;8(6):e019435. doi: 10.1136/bmjopen-2017-019435 (PMC6042563; doi:10.1136/bmjopen-2017-019435)
Supplement: Author's manuscript [file bmjopen-2017-019435.draft_revisions.pdf]

BMJ Open

BMJ Open is committed to open peer review. As part of this commitment we make the peer review history of every article we publish publicly available.

When an article is published we post the peer reviewers' comments and the authors' responses online. We also post the versions of the paper that were used during peer review. These are the versions that the peer review comments apply to.

The versions of the paper that follow are the versions that were submitted during the peer review process. They are not the versions of record or the final published versions. They should not be cited or distributed as the published version of this manuscript.

BMJ Open is an open access journal and the full, final, typeset and author-corrected version of record of the manuscript is available on our site with no access controls, subscription charges or pay-per-view fees (http://bmjopen.bmj.com).

If you have any questions on BMJ Open's open peer review process please email
info.bmjopen@bmj.com

BMJ Open

Acute kidney injury in the UK: temporal and geographical variation in three regions

Journal:	*BMJ Open*
Manuscript ID	bmjopen-2017-019435
Article Type:	Research
Date Submitted by the Author:	01-Sep-2017
Complete List of Authors:	Sawhney, Simon; University of Aberdeen, Insititute of Applied Health Sciences<br>Robinson, Heather; University of Manchester, Centre for Health Informatics, Division of Informatics, Imaging and Data Sciences, Faculty of Biology, Medicine and Health, Manchester Academic Health Science Centre<br>van der Veer, Sabine N.; University of Manchester, Centre for Health Informatics, Division of Informatics, Imaging and Data Sciences, Faculty of Biology, Medicine and Health, Manchester Academic Health Science Centre<br>Hounkpatin, Hilda; University of Southampton, Academic Unit of Primary Care and Population Sciences, Faculty of Medicine<br>Scale, Timothy; Abertawe Bro Morgannwg University Health Board<br>Chess, James; Abertawe Bro Morgannwg University Health Board<br>Peek, N; University of Manchester, Centre for Health Informatics, Division of Informatics, Imaging and Data Sciences, Faculty of Biology, Medicine and Health, Manchester Academic Health Science Centre<br>Marks, Angharad; University of Aberdeen, Insititute of Applied Health Sciences<br>Davies, Gareth; Abertawe Bro Morgannwg University Health Board<br>Fraccaro, Paolo; University of Manchester, Centre for Health Informatics, Division of Informatics, Imaging and Data Sciences, Faculty of Biology, Medicine and Health, Manchester Academic Health Science Centre<br>Johnson, Matthew; University of Southampton, NIHR CLAHRC Wessex Data Science Hub, Faculty of Health Sciences<br>Lyons, Ronan; Abertawe Bro Morgannwg University Health Board<br>Nitsch, Dorothea; London School of Hygiene and Tropical Medicine<br>Roderick, Paul; University of Southampton, Academic Unit of Primary Care and Population Sciences, Faculty of Medicine<br>Halbesma, Nynke; University of Edinburgh, Usher Institute of Population Health Sciences and Informatics<br>Miller-Hodges, Eve; University of Edinburgh, British Heart Foundation Centre for Cardiovascular Science<br>Black, Corrinda; University of Aberdeen, Insititute of Applied Health Sciences<br>Fraser, Simon; University of Southampton, Academic Unit of Primary Care and Population Sciences, Faculty of Medicine
Keywords:	EPIDEMIOLOGY, Nephrology < INTERNAL MEDICINE, Acute renal failure < NEPHROLOGY, PUBLIC HEALTH

For peer review only

Acute kidney injury in the UK: temporal and geographical variation in three regions

Short title:

Variation in acute kidney injury in the UK

Authors:

Dr Simon Sawhney MBChB[1*]; Dr Heather A. Robinson PhD[2*]; Dr Sabine N. van der Veer PhD[2*]; Dr Hilda O. Hounkpatin PhD[3*]; Dr Timothy M. Scale MBChB[4*]; Dr James A. Chess MBChB[4*]; Prof Niels Peek PhD[2*]; Dr Angharad Marks MBChB[1*]; Mr Gareth I. Davies MSc[4*]; Dr Paolo Fraccaro PhD[2*]; Mr Matthew J. Johnson BA[3*]; Prof Ronan A. Lyons MD[4*]; Prof Dorothea Nitsch MD[5*]; Prof Paul J. Roderick MD[6*]; Dr Nynke Halbesma PhD[7*]; Dr Eve Miller-Hodges MBChB[8*]; Prof Corri Black MBChB[1*] and Dr Simon D.S. Fraser DM[6*]

1. Institute of Applied Health Sciences, University of Aberdeen, Aberdeen, UK
2. Centre for Health Informatics, Division of Informatics, Imaging and Data Sciences, Faculty of Biology, Medicine and Health, Manchester Academic Health Science Centre, The University of Manchester, Manchester, UK.
3. NIHR CLAHRC Wessex Data Science Hub, Faculty of Health Sciences, University of Southampton, Southampton, UK
4. Abertawe Bro Morgannwg University Health Board, Swansea, UK
5. London School of Hygiene and Tropical Medicine, London, UK
6. Academic Unit of Primary Care and Population Sciences, Faculty of Medicine, University of Southampton, Southampton, UK
7. Usher Institute of Population Health Sciences and Informatics, University of Edinburgh, Edinburgh, UK
8. British Heart Foundation Centre for Cardiovascular Science, University of Edinburgh, Edinburgh, UK

* The Farr Institute of Health Informatics Research

For peer review only - http://bmjopen.bmj.com/site/about/guidelines.xhtml

Correspondence to:

Simon Sawhney; Institute of Applied Health Sciences; Room 1:040 Polwarth building; Foresterhill;

Aberdeen; AB25 2ZD; simon.sawhney@abdn.ac.uk; +44 (0)1224 437130

For peer review only

For peer review only - http://bmjopen.bmj.com/site/about/guidelines.xhtml

Abstract

Objectives

A rapid growth in the reported rates of acute kidney injury (AKI) has led to calls for greater attention and resources for improving care. However, the reported incidence of acute kidney injury (AKI) also varies more than tenfold between previous studies. Some of this variation is likely to stem from methodological heterogeneity. This study explores the extent of cross-population variation in AKI incidence after minimising heterogeneity.

Design

Population-based cohort study analysing data from electronic health records from three regions in the UK through shared analysis code and harmonised methodology.

Setting

Three populations from Scotland, Wales and England covering three time periods: Grampian 2003, 2007, 2012; Swansea 2007; and Salford 2012.

Participants

All residents in each region, aged 15 years or older.

Main outcome measures

Population incidence of AKI, and AKI phenotype (severity, recovery, recurrence). Determined using shared biochemistry-based AKI episode code and standardised by age and sex.

Results

Respectively, crude AKI rates (per 10,000/year) were: 131, 138, 139, 151 and 124 (p value = 0.095); and after standardisation for age and sex: 147, 151, 146, 146 and 142 (p value = 0.257) for Grampian

For peer review only - http://bmjopen.bmj.com/site/about/guidelines.xhtml

2003, 2007, 2012; Swansea 2007; and Salford 2012. The pattern of variation in crude rates was robust to any modifications of the AKI definition. Across all populations and time periods AKI rates increased substantially with age from ~20 to ~550 per 10,000/year among those aged <40 and ≥70 years.

Conclusion

When harmonised methods are used and age and sex differences are accounted for, a similar high burden of AKI is consistently observed across different populations and time periods (~150 per 10,000/year). There are particularly high rates of AKI among older people. Policy-makers should be careful not draw simplistic assumptions about variation in AKI rates based on comparisons that are not rigorous in methodological terms.

Strengths and limitations

- Previous studies have reported substantial variation in the incidence of AKI between regions and over time, but have involved heterogeneous methods that limit comparability. To our best knowledge, this is the first cross-population study of AKI incidence within one study, with minimised methodological heterogeneity by sharing analysis code across regions.
- By using consistent methods, we provide new evidence that the rates of AKI in the UK are similar across different regions and time periods: ~150 events per 10,000/year (1.5% of the population).
- These findings may not be generalisable outside of the regions of the UK in the study. However to enable researchers to replicate this work, we have made publically available our analysis code for identifying and characterising AKI episodes.

For peer review only - http://bmjopen.bmj.com/site/about/guidelines.xhtml

Introduction

The reported outcomes following acute kidney injury (AKI) are consistently poor[1]. Reports of a growth in rates of AKI have led to calls for greater attention and resources for improving care[2], but there is a more than tenfold variation between studies in the reported population incidence of AKI[3-7]. Population based estimates of AKI incidence range from 18 per 10,000/year[3] to 250 per 10,000/year[5] based on changes in serum creatinine over time, and from 3 to 40 per 10,000/year based on hospital episode codes for "non-dialysis requiring AKI"[8,9]. This wide variation is difficult to fully explain[10], but is likely to be due in part to a changing clinical landscape with evolving international AKI criteria[11-14], and different pragmatic interpretations of AKI criteria in research[5,15]. These reasons for variation are all potential sources of bias in clinical studies of AKI (figure 1). Without a clearer understanding of why populations differ, it is challenging (and potentially misleading) to interpret clinical research in context, to make comparisons across populations or over time, or to make informed public health recommendations.

Worldwide, health services are undertaking quality initiatives to increase clinical awareness and improve treatment of AKI[16-19] in order to achieve the International Society of Nephrology (ISN) target of eliminating avoidable deaths from AKI by 2025[20]. To evaluate the effectiveness of these initiatives, it is vital that there is a harmonisation of approaches to clinical research. This means minimising methodological heterogeneity so that the findings of future research are more comparable, and maximising transparency so that trends in disease incidence and outcomes can be understood. Methodological heterogeneity can arise when researchers extract data from different data infrastructures, make different assumptions, and adopt different criteria for identifying events. These steps are particularly important in AKI, because of the recognised challenges of AKI research: it occurs unpredictably, in different clinical locations[21], may be transient[22], and relies on trends rather than absolute values[12-14]. Small differences in how these challenges are handled can alter both the reported incidence and prognosis of AKI[5,15,21,23]. Despite its importance, this information is

For peer review only - http://bmjopen.bmj.com/site/about/guidelines.xhtml

often undocumented or described in insufficient detail for research to be reproduced[24]. We have described these reasons for variation in AKI rates in a conceptual model (figure 1).

Algorithms using blood test data from electronic health records (EHR), offer the potential of an objective common language for observing common diseases in clinical practice, audit and research[25]. In previous work, we developed an extended version of a widely used NHS algorithm for detecting AKI in blood tests[26] which not only flags individual "AKI" blood tests, but also applies phenotyping methods to combine AKI flagged blood tests into clinically meaningful AKI illness episodes grouped by severity, duration, recovery and recurrence[27,28]. Sharing this algorithm between researchers working with different populations provides an opportunity to develop a harmonised approach to clinical research, robustly comparing the burden of AKI across different populations and over time, even when patient-level data cannot be shared. We used this to study of variation in the incidence of AKI across three populations from England, Scotland and Wales. The analysis spans a decade of change in the clinical awareness of AKI[16], change in international AKI criteria[12-14] and change in the emphasis on community surveillance of people with chronic diseases[29-31]. Our aim was to explore the extent of cross-population variation in AKI incidence while minimising heterogeneity through harmonised methods.

Materials and Methods

Population profiles

This study compares datasets created using linked EHR data from primary and secondary care for three UK regions with different "index" years from 2003-2014: Grampian 2003, 2007 and 2012; Abertawe Bro Morgannwg University Health Board (ABMU, referred to in this article as Swansea) 2007; and Salford 2012 (supplemental figure 1). Each dataset involves health data from the UK NHS and includes complete primary and secondary care biochemistry capture for the region. A fourth

For peer review only - http://bmjopen.bmj.com/site/about/guidelines.xhtml

region initially considered for this analysis (from South England) was excluded because initial inspection of the data characteristics revealed that the population capture of the data source was incomplete and might have led to bias in the estimation of AKI.

NHS Grampian, a health authority in Scotland, is served primarily by one large tertiary hospital and another district general hospital. All biochemistry for the dataset was extracted from a single biochemistry department covering the entire regional population[5]. The Grampian dataset was linked with the Scottish Renal Registry to exclude those already receiving chronic renal replacement therapy (RRT), to avoid misclassification of RRT as AKI. Similarly, Salford (North England) represents one borough of Greater Manchester, served by a single NHS hospital and biochemistry laboratory[32]. Read codes (version 2) were used to extract biochemistry information and exclude records from people receiving chronic RRT. In contrast, ABMU (Swansea, Wales) in 2007 covered a region served by four district general hospitals and four laboratories using two information management systems[33,34]. Those receiving chronic RRT could not be directly determined from a register but could be excluded based on the hospital location marked on the blood tests.

To provide further contextual description of these populations we collected information on population mortality and relevant morbidities (renal and vascular) from the Office of National Statistics, UK Renal Registry, and Quality Outcomes Framework (QOF) data entered by GP practices (table 1). The QOF data represent incentivised recording by GPs of people with a given condition (e.g. chronic kidney disease), rather than actual population prevalences.

Conceptual framework

In figure 1, we provide a conceptual framework for understanding the sources of variation in AKI revealed by our analysis. We sought to minimise "artefactual" methodological differences in AKI episode rates by utilising only datasets where complete data capture (from both hospital and

For peer review only - http://bmjopen.bmj.com/site/about/guidelines.xhtml

community settings) was possible; by harmonising data preparation and cleaning; and by standardising code sets for identifying AKI episodes. We also accounted for "real" potential sources of variation in AKI rates by performing age and sex standardisation, stratification by baseline eGFR for case-mix differences, and comparing the number of people with blood tests in rapid succession as a surrogate for presence of an acute illness.

Data extraction and processing

This study used a distributed analysis approach to protect the confidentiality of patient-level data. Data were analysed by on-site researchers working from the same code. Non-disclosive summary statistics were aggregated into a single dataset, which was analysed centrally. This ensured that patient-level data were never brought together in a single physical location. All serum creatinine results for each individual were extracted. Creatinine values that were missing, were a non-value (e.g. "sample inadequate", "sample error"), or were lower than the limit for detection of the analyser were excluded. The "Modification of Diet in Renal Disease" (MDRD) study estimated glomerular filtration rate (eGFR) was calculated using the abbreviated 4 variable equation[35]. Finally, to avoid a non-chronological evaluation of samples from different locations, where multiple samples were available for the same individual on a given day, the sample with the highest creatinine value was retained for analysis.

AKI identification and phenotyping

Kidney Disease Improving Global Outcomes (KDIGO)-based AKI detection and phenotyping algorithm code was applied by separate analysts working locally on each dataset[14]. As summarised in supplemental table 1, these criteria compare each blood test with previous "baseline" results within the last 365 days ("the look-back period") to determine if a recent change has occurred[27]. Where AKI occurred, a "look-forward period" of 90 days was used to follow and phenotype the whole AKI episode. In supplemental figure 2, these look-back and look-forward time periods are illustrated for

For peer review only - http://bmjopen.bmj.com/site/about/guidelines.xhtml

a single hypothetical patient with respect to a moment of developing AKI within the index year. For those without AKI, the first eGFR of the index year was used as the baseline eGFR. For convenience we used a baseline eGFR <60 ml/min/1.72m^2 as an indicator of chronic kidney disease. Shared Stata code provided the following outputs: number of blood tests consistent with AKI, number of AKI episodes, baseline eGFR, AKI episode severity stage, progression of AKI severity from a lower to higher stage, recovery to baseline within 90 days, and presence of prior AKI episodes in the past three years (i.e. making the episode a recurrent AKI episode).

We also analysed data using more parsimonious versions of the KDIGO criteria: a “narrow interpretation” in which blood tests were only compared if they were no more than a week apart (i.e. restricted to criteria 2 and 3), and a “very narrow interpretation” comparing only tests no more than two days apart (i.e. restricted to criterion 3). If variation was due to a lack of robustness of AKI criteria in the face of estimating baseline from less recent data, these narrower interpretations would be expected to lead to less variation in AKI incidence.

To ensure uniformity of the application and interpretation of AKI code, a mock dataset of 40 hypothetical patients was developed. This mock dataset deliberately contained unformatted variables and a variety of creatinine trend patterns to represent a full range of data cleaning steps, AKI phenotypes, blood test intervals and interpretation issues. Each analyst used the same code on the test dataset and reproduced the same results before progressing to analysing regional data. We have made the algorithm code, mock dataset, and instructions for their optimal use in Stata freely available from https://github.com/RenalHDRUK.

Statistical analysis

Analyses included the description of baseline characteristics, comparison of both crude and age-sex standardised rates of AKI, and phenotypes of AKI episodes. We also compared AKI rates in

For peer review only - http://bmjopen.bmj.com/site/about/guidelines.xhtml

subgroups of baseline eGFR (as described above) and individual components of AKI criteria to determine if variations in rate were robust to changes in the AKI definition (table 2). Finally, to evaluate reasons for residual variation, we described the patterns of blood testing in each region, including the frequency of blood tests, the regularity (e.g. blood tests no more than 2 and 7 days apart) and blood test location (hospital and outpatient/community).

Baseline characteristics included age, sex, the number of people with evidence of renal impairment (eGFR < 60 ml/min/1.73m^2) on their first test in the index year, the number of people with blood tests sufficiently close together for it to be possible to detect an "AKI" result if present (two tests no more than 365 days apart).

We compared population rates of AKI episodes across each region and index year. We compared AKI episode rates using national statistics mid-year population estimates for each region, and then standardised to the England population for 2012[36], a reference population selected as two of the three regions provided 2012 data. All AKI episodes in the index year counted towards the overall AKI episode rate. One way ANOVA followed by Tukey's post-hoc test (in the event of significant differences) was used to identify pairwise significant differences in population level AKI episode rates.

For people with sufficient blood tests to potentially detect an episode of AKI (at least two tests no more than 365 days apart), we compared rates within eGFR strata (<30, 30-44, 45-59 and ≥60 ml/min/1.73m^2). The proportion of the population with at least one AKI result based on AKI criteria 1, 2, or 3 (table 2), and the proportion of the population with at least one AKI result based on narrower interpretations of KDIGO criteria (restricting to criteria 2 & 3, or criterion 3 alone) were also recorded. To evaluate the impact of incomplete biochemistry capture, we also recalculated AKI

For peer review only - http://bmjopen.bmj.com/site/about/guidelines.xhtml

rates using only tests taken from people in hospital. Of note a distinction between hospital inpatient and outpatient results was not possible in Salford

To evaluate potential sources of residual variation in AKI rates after harmonised analysis we compared patterns of blood testing (number, frequency and location).

Patient involvement

No patients were involved in development of the research question or the design of the study. There are no plans to disseminate the results of the research to study participants.

Results

Populations and baseline characteristics

As described in table 1, populations ranged in size from 193,882 (Salford 2012) to 482,444 people (Grampian 2012) (table 3). Crude reported population mortality rates were higher in Swansea than Grampian and Salford, as was the incidence of people starting long term RRT. The recognition of diabetes and cardiovascular diseases in incentivised GP registers was similar across the populations.

Table 2 shows the baseline characteristics of extracted datasets after harmonised data cleaning. The percentage of people with at least two tests no more than 365 days apart varied from 17 – 25% with the fewest in the earliest dataset (Grampian 2003). There was a greater proportion of people tested with renal impairment (estimated glomerular filtration rate, eGFR <60 ml/min/1.73m^2) in 2007 compared to the other years of study.

For peer review only - http://bmjopen.bmj.com/site/about/guidelines.xhtml

Incidence of AKI episodes

Table 3 and figure 2 show the differences in crude and standardised rates of AKI episodes for each dataset. A minority of people had more than one AKI episode in the index year. For reporting AKI episode rates (table 3) all episodes are included, whereas for reporting phenotypes of people with an AKI episode, the first episode is described (bottom of table 3 and table 4). Crude AKI rates varied with the lowest in Salford 2012 and highest rate in Swansea 2007 (124-151 per 10,000/year, p value = 0.095). Standardisation by age and sex accounted for residual differences (142-151 per 10,000/year, p value = 0.257). Age and sex standardised AKI rates varied little between Grampian 2003, 2007 and 2012 (146-151 per 10,000/year).

As shown in figure 3, the pattern of variation in crude AKI rates was the same when narrower interpretations of KDIGO AKI criteria were used, comparing only blood tests in the prior 2 and 7 days. Table 4 shows this pattern was also similar when analysis was limited to each individual component of the AKI criteria, or within strata of baseline eGFR. In addition, across all populations, the proportion of people developing AKI in the index year increased substantially with increasing age and lower eGFR.

AKI phenotypes

Table 4 describes the first AKI episode for people with an AKI episode during the index year. As well as having the highest crude AKI rate, a greater proportion of those with AKI in Swansea were older, had baseline eGFR <60 ml/min/1.73m^2 (37.6%), had a severe AKI episode (15.4% stage 3) had non-recovery at 90 days (45.1%). In Grampian between 2003 and 2012 there was a steady improvement in the proportion of people with renal recovery 90 days after AKI from 42% to 49%.

For peer review only - http://bmjopen.bmj.com/site/about/guidelines.xhtml

Further sources of variation

In addition to assessing for age, sex and case-mix differences, we evaluated the blood testing patterns and clinical location contexts of each dataset (figure 4). Figure 4A shows the frequency of blood tests taken grouped by location: hospital inpatient or outpatient/community. Figure 4B shows the proportion of people with blood tests in close succession. In Grampian from 2003 to 2012, community blood testing increased over time but the frequency of hospital inpatient testing remained unchanged. Test location was not available in Salford, but the proportions of people with two blood tests no more than 2 and 7 days apart was lower than in Grampian and Swansea. Figure 1 shows the conceptual framework for understanding these sources of variation.

Discussion

To our knowledge, this is the first multicentre study to systematically evaluate the extent of and reasons for regional and temporal variation in population rates of AKI, using a harmonised methodological approach. There were differences in the crude rates of AKI between datasets, but after accounting for age and sex, standardised rates were strikingly similar (at 140-150 episodes per 10,000/year, or ~1.5% of the population). The consistently high proportion of people aged over 70 developing AKI was also striking (>5%). This analysis shows the importance of both harmonised methods and standardisation for case-mix prior to any between centre comparisons for description of variation in AKI.

Our analysis provides additional insight into previous reports of a rising AKI incidence in studies based on hospital episode codes or differing AKI definitions[10]. Applying the same KDIGO-based AKI definition to data from the same region, over a ten-year span (2003-2012), the standardised AKI rates in Grampian changed little. Notably, this stability was in spite of an increasing frequency of outpatient/community testing in Grampian (whereas the frequency of hospital inpatient testing changed little over the same period). Our analysis also showed a pattern of AKI phenotypes that was

For peer review only - http://bmjopen.bmj.com/site/about/guidelines.xhtml

consistent with case-mix differences between regions. Swansea, which had the highest all-cause population mortality, also had the highest proportion of AKI phenotypes for severity, AKI progression, and non-recovery.

Between population variation in the prevalence of kidney disease has previously been described for CKD in Ireland[37], Germany[38] and Taiwan[39], as have variation *between* European countries[40]. In our analysis, we have now shown that much of the regional variation in AKI between UK regions can be eliminated by harmonising methods, definitions and correcting for age and sex differences. The stability we report in the AKI incidence over multiple time points in a ten year period is contrary to previous studies from the UK and North America[10]. Given the precautions that we took to minimise heterogeneity, it is possible that some differences reported in previous studies represent a methodological artefact (e.g. data capture or case-mix). Consistent with our findings, a recent study of hospital based AKI among people admitted to the Mayo Clinic also found no significant change in AKI rates between 2006-2014 using a consistent creatinine change AKI definition across each year and stratifying by age and sex[41]. Furthermore, in our analysis the pattern of differences in crude AKI rates was robust to modifications of KDIGO criteria using shorter look-back periods.

Our study has caveats common to observational studies, which we have highlighted in a conceptual model that explains the reasons for observed variation in AKI rates (figure 1). In particular, even though we utilised data from three regions with the same social healthcare system (the UK National Health Service), we encountered incomplete population data capture that led to the exclusion of a fourth region from the study. As we note in figure 1, differences in population capture arising out of incomplete data extraction are not necessarily visible to researchers analysing anonymised large datasets. This serves as a critical caution for researchers and policy-makers to avoid making simplistic assumptions that data from different regions are necessarily comparable when they are derived from different sources. We note that while we have used data from GP registers to provide

For peer review only - http://bmjopen.bmj.com/site/about/guidelines.xhtml

contextual information on the populations, these data need to be interpreted carefully as they reflect recording practices in primary care rather than solely disease burden. We also note that while data from three UK regions were included in our study, this is insufficient to describe variation for the whole of the UK and other countries. This article represents a first step towards more harmonised comparisons of AKI across populations. We have shared our code with this article (https://github.com/RenalHDRUK) and now invite researchers working with population datasets in other regions to add to our experience.

In conclusion, our analysis shows the need for a robust methodological approach and recognition of case-mix differences when evaluating between-centre and temporal trends in AKI. The sharing of code is key to this approach and we have made our code from this article available for researchers to use. Using this approach we show strikingly similar rates of AKI across different populations from England, Scotland and Wales over a ten year period. A consistently high burden of AKI is apparent with an estimated 1.5% of the UK population experiencing AKI each year, rising to more than 5% per year in the elderly. Current quality initiatives should adopt these methods or similar methods when evaluating the impact of changes in practice on the burden of AKI.

Contributors

CB, SF, SS and SV conceived the study. AM, GD, HR, HH, MJ and SS contributed to collection of the data. AM, CB, HH, HR, JC, NP, PF, PR, SS, SV, TS and SF contributed to analysis of the data. AM, CB, DN, EMH, GD, HH, HR, JC, MJ, NH, NP, PF, PR, RL, SS, SV, TS and SF contributed to interpretation of the data. SS and SF drafted the manuscript with input from AM, CB, DN, EMH, GD, HH, HR, JC, MJ, NH, NP, PF, PR, RL, SV and TS. All authors approved the final version.

For peer review only - http://bmjopen.bmj.com/site/about/guidelines.xhtml

Acknowledgements

We would like to acknowledge all the data providers who make anonymised data available for research. This study makes use of anonymised data held in the Secure Anonymised Information Linkage (SAIL) system, which is part of the national e-health records research infrastructure for Wales. We thank the Salford Integrated Record (SIR) board for providing us with the 2014 release of the SIR used in this study.

Funding

This work was funded by a grant from the UK's Farr Institute for Health Informatics Research (UKHIRN/XFarrRP001). The Farr Institute is supported by a 10-funder consortium: Arthritis Research UK, the British Heart Foundation, Cancer Research UK, the Economic and Social Research Council, the Engineering and Physical Sciences Research Council, the Medical Research Council, the National Institute of Health Research, the National Institute for Social Care and Health Research (Welsh Assembly Government), the Chief Scientist Office (Scottish Government Health Directorates), and the Wellcome (MRC Grant Nos: CIPHER MR/K006525/1, HeRC MR/K006665/1, London MR/K006584/1, Scotland MR/K007017/1). We also acknowledge the data management support of Grampian Data Safe Haven (DaSH) and the associated financial support of NHS Research Scotland, through NHS Grampian investment in the Grampian DaSH. Work on this project was also part funded by the National Institute for Health Research (NIHR) Collaboration for Leadership in Applied Health Research and Care (CLAHRC) Wessex and Health Care and Research Wales. SS is supported by a research training fellowship from the Wellcome Trust to study the outcomes of acute kidney injury (WT102729/Z/13/Z). The funders of this study had no role in study design; collection, analysis, and interpretation of data; writing the report; or the decision to submit the report for publication.

For peer review only

For peer review only - http://bmjopen.bmj.com/site/about/guidelines.xhtml

Competing Interests

All authors have completed the ICMJE uniform disclosure form at www.icmje.org/coi_disclosure.pdf and declare: DN reports grants from Informatica, for analyses of the National CKD Audit which was tendered by HQIP (funding from NHS Wales and NHS England), outside the submitted work. SS is supported by a research training fellowship from the Wellcome Trust to study the outcomes of acute kidney injury (WT102729/Z/13/Z). No other support from any organisation for the submitted work; no other financial relationships with any organisations that might have an interest in the submitted work in the previous three years; no other relationships or activities that could appear to have influenced the submitted work.

Ethical Approval

Permission for the use of Grampian biochemistry data using routine biochemistry to identify AKI was provided by University of Aberdeen Sponsor, NHS Grampian Caldicott, respective data custodians, NHS Privacy Advisory Committee (ref PAC 33/14), NHS Research and Development Office (project no. 2014RM003) and National Research Ethics Service (reference 14/NW/1371). Permission to analyse SIR data was granted to North West eHealth via the SIR approval board in 2012, which incorporates the appropriate information governance. Further ethical approval was not required, due to the anonymised nature of the data. We thank the SIR board for providing us with the 2014 release of the SIR used in this study. Permissions for using the SAIL databank were gained through application for the SAIL 0505 project, looking at acute kidney injury in Wales. This was reviewed by the Information governance review panel (IGRP) which contains members of the British Medical Association (BMA), National Research Ethics Service (NRES), public health Wales, NHS Wales informatics service (NWIS) and consumer panel.

For peer review only - http://bmjopen.bmj.com/site/about/guidelines.xhtml

For peer review only

Data Sharing

No additional data available. Analysis code is freely available from https://github.com/RenalHDRUK.

Transparency declaration

The lead author (SS) affirms that the manuscript is an honest, accurate, and transparent account of the study being reported; that no important aspects of the study have been omitted; and that any discrepancies from the study as planned have been explained.

References

[1] Chertow GM, Burdick E, Honour M, Bonventre JV, Bates DW. Acute Kidney Injury, Mortality, Length of Stay, and Costs in Hospitalized Patients. Journal of the American Society of Nephrology 2005;16(11):3365-3370.

[2] Hsu RK, Siew ED. The growth of AKI: half empty or half full, it's the size of the glass that matters. Kidney international 2017;92(3):550-553.

[3] Ali T, Khan I, Simpson W, Prescott G, Townend J, Smith W, et al. Incidence and Outcomes in Acute Kidney Injury: A Comprehensive Population-Based Study. Journal of the American Society of Nephrology 2007;18(4):1292-1298.

[4] Hsu C, McCulloch C, Fan D, Ordonez J, Chertow G, Go A. Community-based incidence of acute renal failure. Kidney international 2007;72(2):208-212.

[5] Sawhney S, Fluck N, Marks A, Prescott G, Simpson W, Tomlinson L, et al. Acute kidney injury—how does automated detection perform? Nephrology Dialysis Transplantation 2015;30(11):1853-1861.

[6] Waikar SS, Curhan GC, Wald R, McCarthy EP, Chertow GM. Declining mortality in patients with acute renal failure, 1988 to 2002. Journal of the American Society of Nephrology 2006;17(4):1143-1150.

[7] Bedford M, Stevens P, Wheeler T, Farmer C. What is the real impact of acute kidney injury? BMC Nephrology 2014;15(1):95.

[8] Kolhe NV, Muirhead AW, Wilkes SR, Fluck RJ, Taal MW. The epidemiology of hospitalised acute kidney injury not requiring dialysis in England from 1998 to 2013: retrospective analysis of hospital episode statistics. International journal of clinical practice 2016;70(4):330-339.

For peer review only - http://bmjopen.bmj.com/site/about/guidelines.xhtml

[9] Hayward JS, McArthur E, Nash DM, Sontrop JM, Russell SJ, Khan S, et al. Kidney Disease Among Registered Métis Citizens of Ontario: A Population-Based Cohort Study. Canadian Journal of Kidney Health and Disease 2017;4:2054358117703071.

[10] Siew ED, Davenport A. The growth of acute kidney injury: a rising tide or just closer attention to detail? Kidney international 2015;87(1):46-61.

[11] Van Biesen W, Vanholder R, Lameire N. Defining Acute Renal Failure: RIFLE and Beyond. Clinical Journal of the American Society of Nephrology 2006;1(6):1314-1319.

[12] Bellomo R, Ronco C, Kellum J, Mehta R, Palevsky P, the Aw. Acute renal failure - definition, outcome measures, animal models, fluid therapy and information technology needs: the Second International Consensus Conference of the Acute Dialysis Quality Initiative (ADQI) Group. Critical Care 2004;8(4):R204-R212.

[13] Mehta RL, Kellum JA, Shah SV, Molitoris BA, Ronco C, Warnock DG, et al. Acute Kidney Injury Network: report of an initiative to improve outcomes in acute kidney injury. Critical care 2007;11(2):R31.

[14] Kidney Disease: Improving Global Outcomes (KDIGO) Acute Kidney Injury Work Group. KDIGO Clinical Practice Guideline for Acute Kidney Injury. Kidney international 2012;2:1-138.

[15] Lafrance JP, Miller DR. Defining acute kidney injury in database studies: the effects of varying the baseline kidney function assessment period and considering CKD status. American Journal of Kidney Diseases 2010;56(4):651-660.

[16] Stewart J, Findlay G, Smith N, Kelly K, Mason M. Adding Insult to Injury: A review of the care of patients who died in hospital with a primary diagnosis of acute kidney injury. London: National Confidential Inquiry into Patient Outcome and Death.; 2009.

[17] Silver SA, Adu D, Agarwal S, Gupta KL, Lewington AJP, Pannu N, et al. Strategies to Enhance Rehabilitation After Acute Kidney Injury in the Developing World. Kidney International Reports 2017;2(4):579-593.

[18] Think Kidneys. Think Kidneys. 2015; Available at: https://www.thinkkidneys.nhs.uk/. Accessed 07/04, 2017.

[19] Mehta R, Bagga A, Patibandla R, Chakravarthi R. Detection and Management of AKI in the Developing World: The 18th Acute Disease Quality Initiative (ADQI) International Consensus Conference. Kidney International Reports 2017;2(4):515-518.

[20] Mehta RL, Cerda J, Burdmann EA, Tonelli M, Garcia-Garcia G, Jha V, et al. International Society of Nephrology's 0by25 initiative for acute kidney injury (zero preventable deaths by 2025): a human rights case for nephrology. Lancet 2015;385(9987):2616-2643.

[21] Sawhney S, Fluck N, Fraser SD, Marks A, Prescott GJ, Roderick PJ, et al. KDIGO-based acute kidney injury criteria operate differently in hospitals and the community—findings from a large population cohort. Nephrology Dialysis Transplantation 2016;31(6):922-929.

[22] Uchino S, Bellomo R, Bagshaw SM, Goldsmith D. Transient azotaemia is associated with a high risk of death in hospitalized patients. Nephrology Dialysis Transplantation 2010;25(6):1833-1839.

For peer review only - http://bmjopen.bmj.com/site/about/guidelines.xhtml

[23] Sawhney S, Mitchell M, Marks A, Fluck N, Black C. Long-term prognosis after acute kidney injury (AKI): what is the role of baseline kidney function and recovery? A systematic review. BMJ Open 2015;5(1):e006497.

[24] Playford CJ, Gayle V, Connelly R, Gray AJG. Administrative social science data: The challenge of reproducible research. Big Data & Society 2016;3(2):2053951716684143.

[25] Bagshaw SM, Goldstein SL, Ronco C, Kellum JA. Acute kidney injury in the era of big data: the 15th Consensus Conference of the Acute Dialysis Quality Initiative (ADQI). Canadian Journal of Kidney Health and Disease 2016;3(1):1-5.

[26] NHS England. Patient safety alert on standardising the early identification of Acute Kidney Injury. 2014; Available at: http://www.england.nhs.uk/2014/06/09/psa-aki/. Accessed 07/04, 2017.

[27] Sawhney S, Marks A, Fluck N, Levin A, Prescott GJ, Black C. Intermediate and Long-Term Outcomes of Survivors of Acute Kidney Injury Episodes: A Large Population-Based Cohort Study. American Journal of Kidney Diseases 2017;69(1):18-28.

[28] Sawhney S, Marks A, Fluck N, Levin A, McLernon D, Prescott G, et al. Post-discharge kidney function is associated with subsequent ten-year renal progression risk among survivors of acute kidney injury. Kidney international 2017;92(2):440-452.

[29] Kidney Disease: Improving Global Outcomes (KDIGO) CKD Work Group. KDIGO Clinical Practice Guideline for the Evaluation and Management of Chronic Kidney Disease. Kidney international 2013;3:1-150.

[30] Yancy CW, Jessup M, Bozkurt B, Butler J, Casey DE, Drazner MH, et al. 2013 ACCF/AHA Guideline for the Management of Heart Failure. Circulation 2013;128(16):e240.

[31] Guideline Development Group, Bilo H, Coentrao L, Couchoud C, Covic A, De Sutter J, et al. Clinical Practice Guideline on management of patients with diabetes and chronic kidney disease stage 3b or higher (eGFR <45 mL/min). Nephrology Dialysis Transplantation 2015;30:ii1-ii142.

[32] Fraccaro P, van der Veer SN, Brown B, Prosperi M, O'Donoghue D, Collins GS, et al. An external validation of models to predict the onset of chronic kidney disease using population-based electronic health records from Salford, UK. BMC Medicine 2016;14(1):104.

[33] Ford DV, Jones KH, Verplancke J, Lyons RA, John G, Brown G, et al. The SAIL Databank: building a national architecture for e-health research and evaluation. BMC Health Services Research 2009;9(1):157.

[34] Lyons RA, Jones KH, John G, Brooks CJ, Verplancke J, Ford DV, et al. The SAIL databank: linking multiple health and social care datasets. BMC Medical Informatics and Decision Making 2009;9(1):3.

[35] Levey AS, Stevens LA, Schmid CH, Zhang Y, Castro AF, Feldman HI, et al. A New Equation to Estimate Glomerular Filtration Rate. Annals of Internal Medicine 2009;150(9):604-612.

[36] Roalfe AK, Holder RL, Wilson S. Standardisation of rates using logistic regression: a comparison with the direct method. BMC Health Services Research 2008;8(1):275.

For peer review only - http://bmjopen.bmj.com/site/about/guidelines.xhtml

[37] Stack AG, Casserly LF, Cronin CJ, Chernenko T, Cullen W, Hannigan A, et al. Prevalence and variation of Chronic Kidney Disease in the Irish health system: initial findings from the National Kidney Disease Surveillance Programme. BMC Nephrology 2014;15(1):185.

[38] Aumann N, Baumeister SE, Rettig R, Lieb W, Werner A, Doring A, et al. Regional Variation of Chronic Kidney Disease in Germany: Results From Two Population-Based Surveys. Kidney and Blood Pressure Research 2015;40(3):231-243.

[39] Yap Y, Chuang K, Chiang C, Chuang H, Lu S. Geographic Variation of Chronic Kidney Disease Prevalence: Correlation with the Incidence of Renal Cell Carcinoma or Urothelial Carcinoma? BioMed Research International 2015;2015:427084.

[40] Brück K, Stel VS, Gambaro G, Hallan S, Völzke H, Ärnlöv J, et al. CKD Prevalence Varies across the European General Population. Journal of the American Society of Nephrology 2016;27(7):2135-2147.

[41] Kashani K, Shao M, Li G, Williams AW, Rule AD, Kremers WK, et al. No increase in the incidence of acute kidney injury in a population-based annual temporal trends epidemiology study. Kidney international 2017;(in press).

For peer review only

For peer review only - http://bmjopen.bmj.com/site/about/guidelines.xhtml

Tables and Figures

Table 1 – Contextual information on the populations in this study

	Grampian 2003	Grampian 2007	Grampian 2012	Swansea 2007	Salford 2012
**Index year for assessing incident AKI episodes**	2003	2007	2012	2007	2012
**Mid-year regional population (all ages) during index year[1]**	529,360	548,290	573,400	499,400	237,085
**Mid-year regional adult population (age ≥ 15 years) during index year[1]**	438,332	458,900	482,444	415,500	193,882
**Percentage of population in urban settlements of > 10,000 people[2]**	49.3%	51.8%	52.1%	81.7%	99.9%
**Regional crude all-cause mortality rate ages 15+ (index year/100,000)[1]**	1192	1154	1093	1334	1135
**Crude adult incidence of chronic RRT per million population (UKRR)[3]**	98	102	93	167	85
**Prevalence of chronic kidney disease per 100 people (QOF)[4]**	n/a[5]	2.6	3.3	1.8	3.0
**Prevalence of coronary heart disease per 100 people (QOF)[4]**	4.1	4.0	3.9	4.1	3.9
**Prevalence of diabetes registration per 100 people (QOF)[4]**	3.0	3.3	4.2	4.3	4.5
**Prevalence of heart failure registration per 100 people (QOF)[4]**	n/a[4]	0.8	0.8	1.0	0.9
**Prevalence of hypertension registration per 100 people (QOF)[4]**	11.0	11.7	13.2	12.5	13.8
**Prevalence of stroke & TIA registration per 100 people (QOF)[4]**	1.6	1.7	1.9	2.1	1.8
**Number of biochemistry departments for whole region**	One department covers in and outpatient, community and private tests	One department covers in and outpatient, community and private tests	One department covers in and outpatient, community and private tests	Four departments cover in and outpatients, community and private tests	One department covers in and outpatient and community tests. Privately obtained samples unavailable
**Means of excluding samples belonging to people on long term RRT from dataset**	Link to Scottish Renal Registry	Link to Scottish Renal Registry	Link to Scottish Renal Registry	Removing samples from locations where renal replacement is performed, including intensive care unit	Read code screening
**IDMS aligned creatinine assay**	Yes	Yes	Yes	From 2007	Yes

[1] From the Office of National Statistics
[2] From the 2011 National Census in England and Wales and Scottish Government Urban Rural Classification
[3] From the UK Renal Registry (UKRR) annual reports
[4] Quality Outcomes Framework (QOF) data is incentivised information entered by GP practices. Not recorded in Grampian in 2003, for which 2004 data is provided where available.
[5] Data not available

For peer review only - http://bmjopen.bmj.com/site/about/guidelines.xhtml

Table 2 – Baseline characteristics for each dataset

	Grampian 2003		Grampian 2007		Grampian 2012		Swansea 2007		Salford 2012	
	Patient total	(%)[1]	Patient total	(%)	Patient total	(%)	Patient total	(%)	Patient total	(%)
Adult resident population (aged ≥ 15)	438332		458900		482444		415500		193882	
**Population ascertainment of renal impairment (eGFR < 60 ml/min/1.73m^2) in index year**										
No tests during index year	311922	(71.2)	303673	(66.2)	301992	(62.6)	253531	(61.0)	116977	(60.3)
eGFR ≥60 [2]	101595	(23.2)	120854	(26.3)	158736	(32.9)	129959	(31.3)	66890	(34.5)
eGFR <60 [2]	24805	(5.7)	34373	(11.3)	21716	(4.5)	32010	(7.7)	10015	(5.2)
**Sufficiency of tests to enable AKI detection**										
People with no tests during index year	311922	(71.2)	303673	(66.2)	301992	(62.6)	253531	(61.0)	116977	(60.3)
People with insufficient tests	52602	(12.0)	57788	(12.6)	69239	(14.4)	59839	(14.4)	31467	(16.2)
People with ≥2 tests within 365 days	73808	(16.8)	97439	(21.2)	111213	(23.1)	102130	(24.6)	45438	(23.4)
**Characteristics of people with ≥ 2 tests within 365 days**										
Proportion female	40413	(54.8)	53061	(54.5)	60330	(54.2)	55685	(54.5)	24723	(54.4)
Median age (IQR)	63	(48-74)	63	(50-75)	63	(49-74)	64	(51-75)	63	(49-74)
eGFR <60 [2]	18573	(25.2)[3]	28274	(29.0)	18679	(20.2)	25952	(25.4)	8541	(18.8)

[1] Expressed as a percentage of total residents unless specified otherwise
[2] First estimated glomerular filtration rate in index year (ml/min/1.73m^2)
[3] Expressed as a percentage of people with ≥ 2 tests within 365 days

For peer review only

For peer review only - http://bmjopen.bmj.com/site/about/guidelines.xhtml

Table 3 – Crude and standardised rates of AKI episodes, and components of AKI criteria

	Grampian 2003 (Rate per 10,000)[1]	Grampian 2007 (Rate per 10,000)	Grampian 2012 (Rate per 10,000)	Swansea 2007 (Rate per 10,000)	Salford 2012 (Rate per 10,000)
Adult resident population	438332	458900	482444	415500	193882
**AKI incidence rates**					
Crude AKI incidence (95% CI)	131.2 (127.7-134.7)	138.3 (134.9-141.7)	139.1 (135.8-142.4)	151.1 (147.4-154.8)	124.3 (118.8-129.8)
Age-sex standardised AKI incidence (95% CI)	147.2 (143.3-151.1)	150.6 (146.9-154.3)	146.3 (142.8-149.8)	145.6 (142.0-149.2)	141.8 (136.2-147.4)
Total AKI episodes	5749 (131)	6346 (138)	6711 (139)	6266 (151)	2399 (124)
People with AKI	5362 (122)	5930 (129)	6277 (130)	5847 (141)	2208 (114)
**Subgroups of people with AKI**					
AKI using hospital tests only	4386 (100)	4739 (103)	4492 (93)	4432 (107)	n/a[2]
Rigid KDIGO criteria	3436 (78)	3803 (83)	3617 (75)	3469 (83)	1114 (57)
People meeting 2d criterion	2486 (57)	2831 (62)	2714 (56)	2424 (58)	741 (38)
People meeting 7d criterion	2488 (57)	2698 (56)	2664 (55)	2611 (63)	821 (42)
People meeting 8-90d criterion	2619 (60)	2830 (59)	3351 (69)	3287 (79)	1163 (60)
People meeting 91-365d criterion	1408 (32)	1528 (32)	1850 (38)	1591 (38)	737 (38)
**People with AKI in age strata**					
≥70 years	3205 (562)	3561 (587)	3705 (572)	3785 (584)	1299 (544)
40-69 years	1765 (88)	1903 (89)	2021 (89)	1699 (89)	740 (92)
<40 years	392 (22)	466 (25)	551 (29)	363 (23)	169 (19)
**People with AKI in eGFR strata among people with at least two tests within 365 days (rates expressed within strata of tested individuals at risk)**					
Baseline eGFR ≥60	3612 (654)	3874 (560)	4419 (478)	3648 (479)	1512 (410)
Baseline eGFR 45-59	809 (673)	940 (496)	894 (756)	1044 (618)	323 (607)
Baseline eGFR 30-44	597 (1222)	723 (1000)	661 (1282)	732 (1097)	202 (867)
Baseline eGFR <30	344 (2064)	393 (1861)	303 (1781)	423 (1778)	171 (1921)

[1]Rate expressed per 10,000 residents unless specified otherwise
[2]Location data not available

For peer review only - http://bmjopen.bmj.com/site/about/guidelines.xhtml

Table 4 – Phenotype of AKI episodes

	Grampian 2003		Grampian 2007		Grampian 2012		Swansea 2007		Salford 2012	
	Total people	(%)	Total people	(%)	Total people	(%)	Total people	(%)	Total people	(%)
**People with AKI**	5362		5930		6277		5847		2208	
Proportion female	2899	(54.1)	3256	(54.9)	3443	(54.9)	3195	(54.6)	1250	(56.6)
Median age (IQR)	73	(61-81)	74	(61-82)	74	(60-82)	76	(64-84)	74	(61-83)
**Peak AKI severity stage for first episode**										
stage 1	3720	(69.4)	4211	(71.0)	4389	(69.9)	3720	(63.6)	1435	(65.0)
stage 2	1014	(18.9)	1063	(17.9)	1174	(18.7)	1224	(20.9)	451	(20.4)
stage 3	628	(11.7)	656	(11.1)	714	(11.4)	903	(15.4)	322	(14.6)
AKI stage progression	817	(15.2)	792	(13.4)	850	(13.5)	900	(15.4)	300	(13.6)
**Baseline eGFR for first episode (ml/min/1.73m^2)**										
≥60	3612	(67.4)	3874	(65.3)	4419	(70.4)	3648	(62.4)	1512	(68.5)
45-59	809	(15.1)	940	(15.9)	894	(14.2)	1044	(17.9)	323	(14.6)
30-44	597	(11.1)	723	(12.2)	661	(10.5)	732	(12.5)	202	(9.1)
<30	344	(6.4)	393	(6.6)	303	(4.8)	423	(7.2)	171	(7.7)
**Prior AKI episodes detected in last 3 years**										
No prior episodes	4415	(82.3)	4847	(81.7)	5052	(80.5)	4824	(82.5)	1708	(77.4)
1 prior episode	723	(13.5)	833	(14.0)	897	(14.3)	784	(13.4)	349	(15.8)
2 or more prior episodes	224	(4.2)	250	(4.2)	328	(5.2)	239	(4.1)	151	(6.8)
Prior AKI within 1 year	414	(7.7)	459	(7.7)	492	(7.8)	488	(8.3)	216	(9.8)
**Renal recovery to within 20% of baseline**										
Renal recovery	2239	(41.8)	2588	(43.6)	3077	(49.0)	2156	(36.9)	970	(43.9)
Renal non-recovery	2203	(41.1)	2387	(40.3)	2245	(35.8)	2635	(45.1)	820	(37.1)
Repeat samples not available	920	(17.2)	955	(16.1)	955	(15.2)	1056	(18.1)	418	(18.9)

[1]Expressed as a percentage of people with at least one AKI episode
[2]Insufficient biochemistry data available to report on the previous 3 years

For peer review only - http://bmjopen.bmj.com/site/about/guidelines.xhtml

Figure 1 – Conceptual framework for the reasons for cross-population differences in AKI rates

Figure 2 – Crude and age-sex standardised rate of AKI episodes

Figure 3 – Crude AKI rates using different interpretations of the KDIGO-based AKI definition

Figure 4 – Patterns of blood testing by clinical location (4A), and by test regularity (4B)

For peer review only

For peer review only - http://bmjopen.bmj.com/site/about/guidelines.xhtml

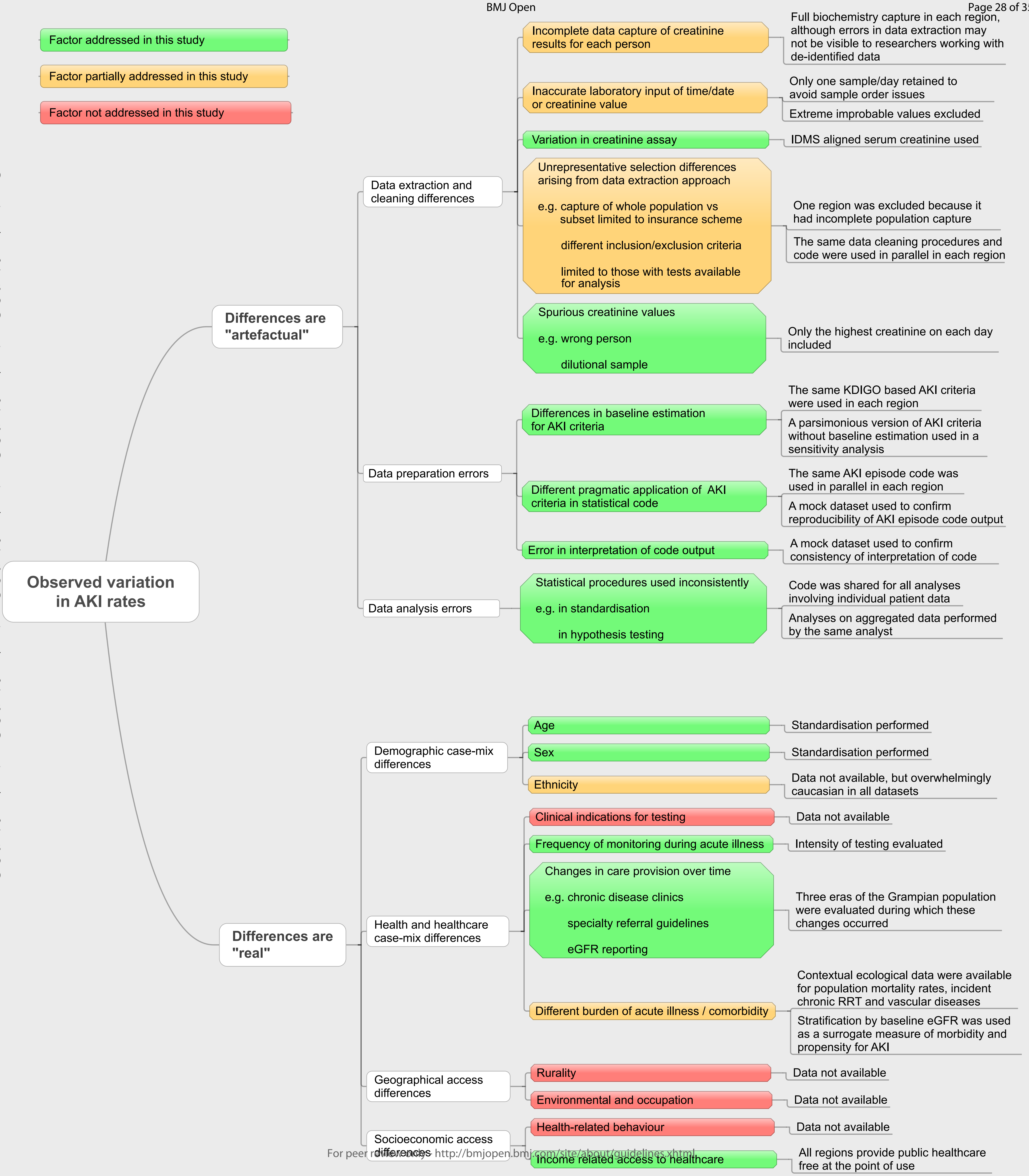
Factor addressed in this study
Factor partially addressed in this study
Factor not addressed in this study
Observed variation in AKI rates
Differences are "artefactual"
Data extraction and cleaning differences
Incomplete data capture of creatinine results for each person
Full biochemistry capture in each region, although errors in data extraction may not be visible to researchers working with de-identified data
Inaccurate laboratory input of time/date or creatinine value
Only one sample/day retained to avoid sample order issues
Extreme improbable values excluded
Variation in creatinine assay
IDMS aligned serum creatinine used
Unrepresentative selection differences arising from data extraction approach
e.g. capture of whole population vs subset limited to insurance scheme
different inclusion/exclusion criteria
limited to those with tests available for analysis
One region was excluded because it had incomplete population capture
The same data cleaning procedures and code were used in parallel in each region
Spurious creatinine values
e.g. wrong person
dilutional sample
Only the highest creatinine on each day included
Data preparation errors
Differences in baseline estimation for AKI criteria
The same KDIGO based AKI criteria were used in each region
A parsimonious version of AKI criteria without baseline estimation used in a sensitivity analysis
Different pragmatic application of AKI criteria in statistical code
The same AKI episode code was used in parallel in each region
A mock dataset used to confirm reproducibility of AKI episode code output
Error in interpretation of code output
A mock dataset used to confirm consistency of interpretation of code
Data analysis errors
Statistical procedures used inconsistently
e.g. in standardisation
in hypothesis testing
Code was shared for all analyses involving individual patient data
Analyses on aggregated data performed by the same analyst
Differences are "real"
Demographic case-mix differences
Age
Standardisation performed
Sex
Standardisation performed
Ethnicity
Data not available, but overwhelmingly caucasian in all datasets
Health and healthcare case-mix differences
Clinical indications for testing
Data not available
Frequency of monitoring during acute illness
Intensity of testing evaluated
Changes in care provision over time
e.g. chronic disease clinics
specialty referral guidelines
eGFR reporting
Three eras of the Grampian population were evaluated during which these changes occurred
Different burden of acute illness / comorbidity
Contextual ecological data were available for population mortality rates, incident chronic RRT and vascular diseases
Stratification by baseline eGFR was used as a surrogate measure of morbidity and propensity for AKI
Geographical access differences
Rurality
Data not available
Environmental and occupation
Data not available
Socioeconomic access differences
Health-related behaviour
Data not available
Income related access to healthcare
All regions provide public healthcare free at the point of use

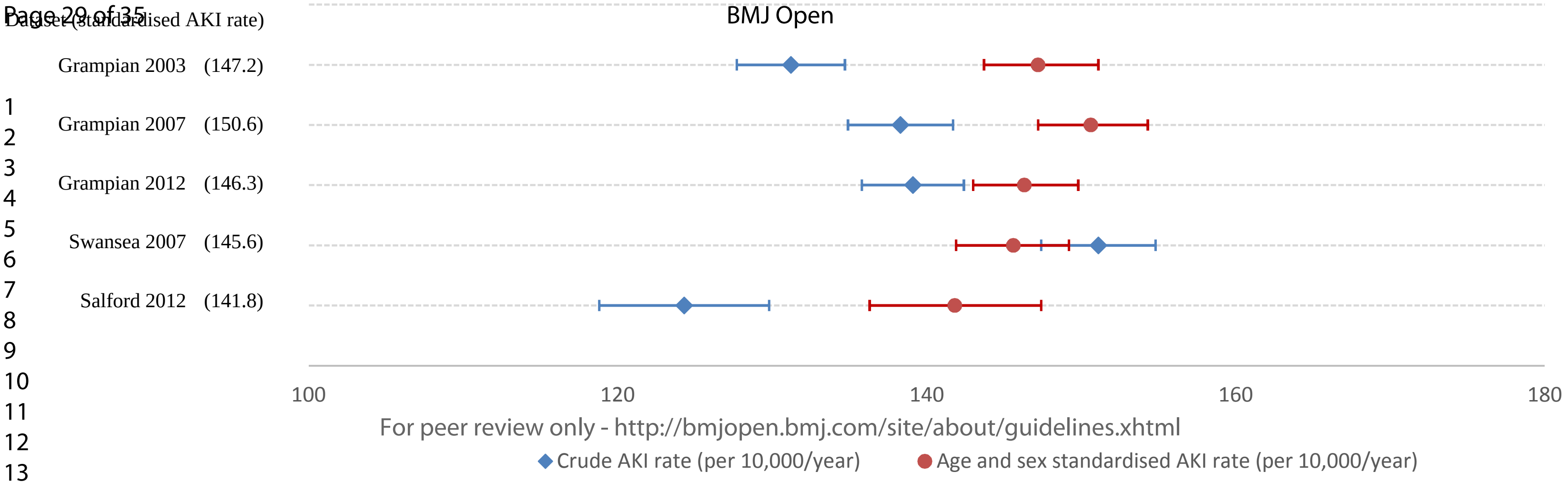
Dataset (standardised AKI rate)
Grampian 2003 (147.2)
Grampian 2007 (150.6)
Grampian 2012 (146.3)
Swansea 2007 (145.6)
Salford 2012 (141.8)
100
120
140
160
180
Crude AKI rate (per 10,000/year)
Age and sex standardised AKI rate (per 10,000/year)

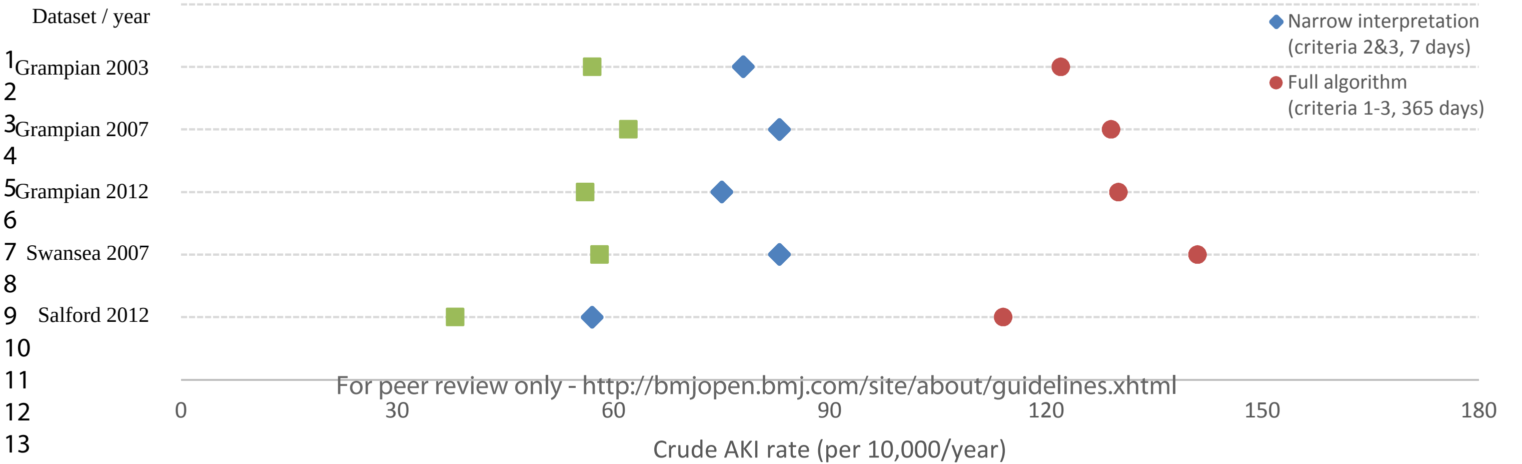

For peer review only - http://bmjopen.bmj.com/site/about/guidelines.xhtml

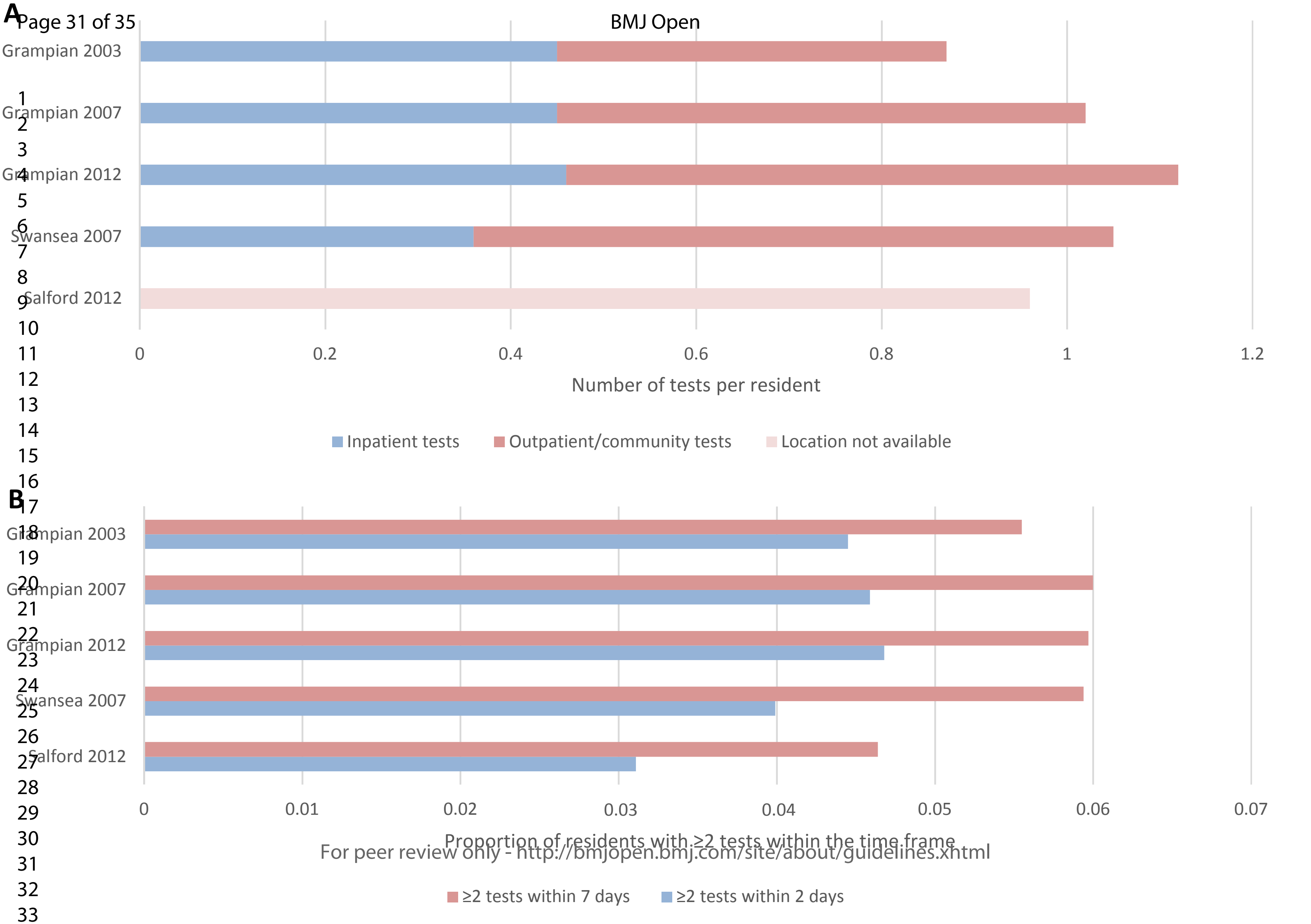
A
Grampian 2003
Grampian 2007
Grampian 2012
Swansea 2007
Salford 2012
0
0.2
0.4
0.6
0.8
1
1.2
Number of tests per resident
Inpatient tests
Outpatient/community tests
Location not available
B
Grampian 2003
Grampian 2007
Grampian 2012
Swansea 2007
Salford 2012
0
0.01
0.02
0.03
0.04
0.05
0.06
0.07
Proportion of residents with ≥2 tests within the time frame
≥2 tests within 7 days
≥2 tests within 2 days

Supplemental table 1 – AKI definition and phenotype criteria for this study

AKI Criteria	AKI definition
Criterion 1	Serum creatinine ≥1.5 times higher than the median of all creatinine values 8-90 days ago, or 91-365 days ago if no tests between 8-90 days
Criterion 2	Serum creatinine ≥1.5 times higher than the lowest creatinine within 7 days
Criterion 3	Serum creatinine >26 µmol/L higher than the lowest creatinine within 48 hours
**AKI severity**	**Staging definition (based on peak creatinine within 90 days of diagnosis)**
Stage 1	Rise in creatinine of >26 µmol/L; or index/baseline ratio ≥1.5 and <2
Stage 2	Index/baseline ratio ≥2 and <3
Stage 3	Index/baseline ratio ≥3; or ≥1.5 and index creatinine >354 µmol/L
**Prior AKI episodes**	**Prior AKI definition**
No prior AKI	AKI episode not preceded by any previous AKI episodes in the prior 3 years
Prior AKI	AKI episode preceded by at least one previous AKI episode in the prior 3 years
Recent prior AKI	AKI episode preceded by at least one previous AKI episode in the prior 1 year
**90 day AKI recovery**	**Recovery definition**
Recovery	Last creatinine within 90 days of AKI <1.2 times higher than the baseline creatinine at diagnosis
Non-recovery	Last creatinine within 90 days of AKI ≥1.2 times higher than the baseline creatinine at diagnosis, or still receiving acute RRT
"Untested"	No repeat blood tests taken within 90 days of AKI diagnosis

Abbreviations: AKI, acute kidney injury; RRT, renal replacement therapy

For peer review only

Supplementary figure 1 – Map of the UK populations in this analysis

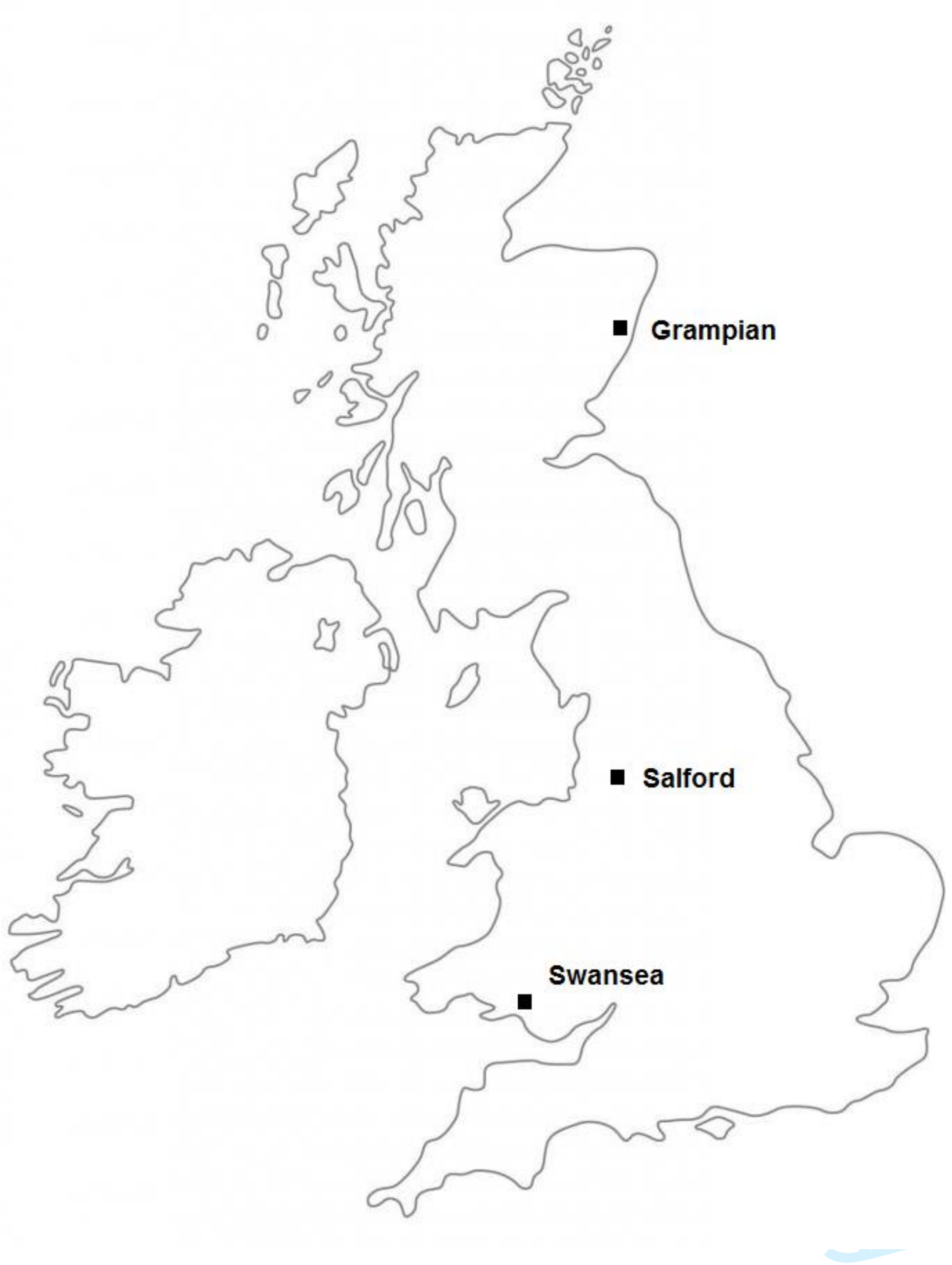

Supplemental figure 2 – Hypothetical patient illustrating look-back (for baseline) and look-forward (for AKI episode phenotyping) time periods from the start of an AKI episode

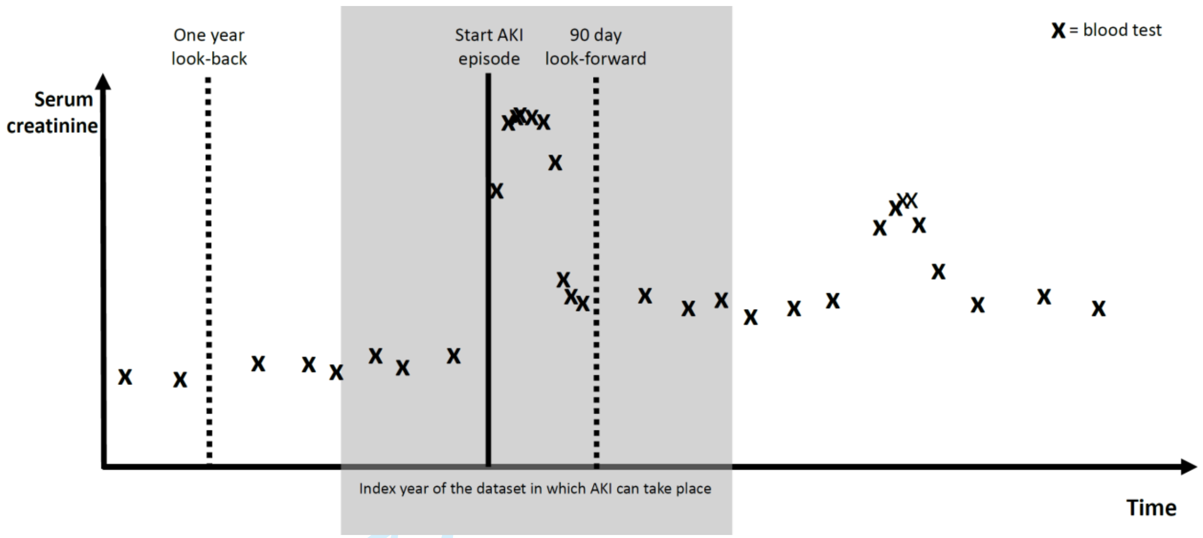

For peer review only

STROBE Statement—Checklist of items that should be included in reports of ***cohort studies***

	Item No	Recommendation	Pages
**Title and abstract**	1	(*a*) Indicate the study's design with a commonly used term in the title or the abstract	2
		(*b*) Provide in the abstract an informative and balanced summary of what was done and what was found	2
**Introduction**			
Background/rationale	2	Explain the scientific background and rationale for the investigation being reported	4
Objectives	3	State specific objectives, including any prespecified hypotheses	5
**Methods**			
Study design	4	Present key elements of study design early in the paper	5
Setting	5	Describe the setting, locations, and relevant dates, including periods of recruitment, exposure, follow-up, and data collection	5
Participants	6	(*a*) Give the eligibility criteria, and the sources and methods of selection of participants. Describe methods of follow-up	5, 6
		(*b*) For matched studies, give matching criteria and number of exposed and unexposed	n/a
Variables	7	Clearly define all outcomes, exposures, predictors, potential confounders, and effect modifiers. Give diagnostic criteria, if applicable	7
Data sources/ measurement	8*	For each variable of interest, give sources of data and details of methods of assessment (measurement). Describe comparability of assessment methods if there is more than one group	7, 8
Bias	9	Describe any efforts to address potential sources of bias	8
Study size	10	Explain how the study size was arrived at	5, 10, table 1
Quantitative variables	11	Explain how quantitative variables were handled in the analyses. If applicable, describe which groupings were chosen and why	9
Statistical methods	12	(*a*) Describe all statistical methods, including those used to control for confounding	9
		(*b*) Describe any methods used to examine subgroups and interactions	9
		(*c*) Explain how missing data were addressed	n/a
		(*d*) If applicable, explain how loss to follow-up was addressed	n/a
		(*e*) Describe any sensitivity analyses	9
**Results**			
Participants	13*	(a) Report numbers of individuals at each stage of study—eg numbers potentially eligible, examined for eligibility, confirmed eligible, included in the study, completing follow-up, and analysed	10, tables 1 & 2
		(b) Give reasons for non-participation at each stage	6
		(c) Consider use of a flow diagram	n/a
Descriptive data	14*	(a) Give characteristics of study participants (eg demographic, clinical, social) and information on exposures and potential confounders	Tables 1 and 2
		(b) Indicate number of participants with missing data for each variable of interest	n/a
		(c) Summarise follow-up time (eg, average and total amount)	n/a
Outcome data	15*	Report numbers of outcome events or summary measures over time	10, 11, table 3

For peer review only - http://bmjopen.bmj.com/site/about/guidelines.xhtml

Main results	16	(*a*) Give unadjusted estimates and, if applicable, confounder-adjusted estimates and their precision (eg, 95% confidence interval). Make clear which confounders were adjusted for and why they were included	11, table 3
		(*b*) Report category boundaries when continuous variables were categorized	Table 3
		(*c*) If relevant, consider translating estimates of relative risk into absolute risk for a meaningful time period	n/a
Other analyses	17	Report other analyses done—eg analyses of subgroups and interactions, and sensitivity analyses	Tables 3 & 4
**Discussion**			
Key results	18	Summarise key results with reference to study objectives	12
Limitations	19	Discuss limitations of the study, taking into account sources of potential bias or imprecision. Discuss both direction and magnitude of any potential bias	13
Interpretation	20	Give a cautious overall interpretation of results considering objectives, limitations, multiplicity of analyses, results from similar studies, and other relevant evidence	14
Generalisability	21	Discuss the generalisability (external validity) of the study results	13
**Other information**			
Funding	22	Give the source of funding and the role of the funders for the present study and, if applicable, for the original study on which the present article is based	14

*Give information separately for exposed and unexposed groups.

Note: An Explanation and Elaboration article discusses each checklist item and gives methodological background and published examples of transparent reporting. The STROBE checklist is best used in conjunction with this article (freely available on the Web sites of PLoS Medicine at http://www.plosmedicine.org/, Annals of Internal Medicine at http://www.annals.org/, and Epidemiology at http://www.epidem.com/). Information on the STROBE Initiative is available at http://www.strobe-statement.org.

For peer review only

For peer review only - http://bmjopen.bmj.com/site/about/guidelines.xhtml

BMJ Open

Acute kidney injury in the UK: A replication cohort study of the variation across three regional populations

Journal:	*BMJ Open*
Manuscript ID	bmjopen-2017-019435.R1
Article Type:	Research
Date Submitted by the Author:	28-Feb-2018

Complete List of Authors:	Sawhney, Simon; University of Aberdeen, Insititute of Applied Health Sciences<br>Robinson, Heather; University of Manchester, Centre for Health Informatics, Division of Informatics, Imaging and Data Sciences, Faculty of Biology, Medicine and Health, Manchester Academic Health Science Centre<br>van der Veer, Sabine N.; University of Manchester, Centre for Health Informatics, Division of Informatics, Imaging and Data Sciences, Faculty of Biology, Medicine and Health, Manchester Academic Health Science Centre<br>Hounkpatin, Hilda; University of Southampton, Academic Unit of Primary Care and Population Sciences, Faculty of Medicine<br>Scale, Timothy; Swansea University<br>Chess, James; Swansea University<br>Peek, N; University of Manchester, Centre for Health Informatics, Division of Informatics, Imaging and Data Sciences, Faculty of Biology, Medicine and Health, Manchester Academic Health Science Centre<br>Marks, Angharad; University of Aberdeen, Insititute of Applied Health Sciences<br>Davies, Gareth; Swansea University<br>Fraccaro, Paolo; University of Manchester, Centre for Health Informatics, Division of Informatics, Imaging and Data Sciences, Faculty of Biology, Medicine and Health, Manchester Academic Health Science Centre<br>Johnson, Matthew; University of Southampton, NIHR CLAHRC Wessex Data Science Hub, Faculty of Health Sciences<br>Lyons, Ronan; Swansea University<br>Nitsch, Dorothea; London School of Hygiene and Tropical Medicine<br>Roderick, Paul; University of Southampton, Academic Unit of Primary Care and Population Sciences, Faculty of Medicine<br>Halbesma, Nynke; University of Edinburgh, Usher Institute of Population Health Sciences and Informatics<br>Miller-Hodges, Eve; University of Edinburgh, British Heart Foundation Centre for Cardiovascular Science<br>Black, Corrinda; University of Aberdeen, Insititute of Applied Health Sciences<br>Fraser, Simon; University of Southampton, Academic Unit of Primary Care and Population Sciences, Faculty of Medicine

**Primary Subject Heading**:	Renal medicine
Secondary Subject Heading:	Public health, Epidemiology
Keywords:	EPIDEMIOLOGY, Nephrology < INTERNAL MEDICINE, Acute renal failure <

	NEPHROLOGY, PUBLIC HEALTH

SCHOLARONE™ Manuscripts

For peer review only

Acute kidney injury in the UK: A replication cohort study of the variation across three regional populations

Short title:

Variation in acute kidney injury in the UK

Authors:

Dr Simon Sawhney MBChB[1*]; Dr Heather A. Robinson PhD[2*]; Dr Sabine N. van der Veer PhD[2*]; Dr Hilda O. Hounkpatin PhD[3*]; Dr Timothy M. Scale MBChB[4*]; Dr James A. Chess MBChB[4*]; Prof Niels Peek PhD[2*]; Dr Angharad Marks MBChB[1*]; Mr Gareth I. Davies MSc[4*]; Dr Paolo Fraccaro PhD[2*]; Mr Matthew J. Johnson BA[3*]; Prof Ronan A. Lyons MD[4*]; Prof Dorothea Nitsch MD[5*]; Prof Paul J. Roderick MD[6*]; Dr Nynke Halbesma PhD[7*]; Dr Eve Miller-Hodges MBChB[8*]; Prof Corri Black MBChB[1*] and Dr Simon D.S. Fraser DM[6*]

1. Institute of Applied Health Sciences, University of Aberdeen, Aberdeen, UK
2. Centre for Health Informatics, Division of Informatics, Imaging and Data Sciences, Faculty of Biology, Medicine and Health, Manchester Academic Health Science Centre, The University of Manchester, Manchester, UK.
3. NIHR CLAHRC Wessex Data Science Hub, Faculty of Health Sciences, University of Southampton, Southampton, UK
4. University of Swansea, Swansea, UK
5. London School of Hygiene and Tropical Medicine, London, UK
6. Academic Unit of Primary Care and Population Sciences, Faculty of Medicine, University of Southampton, Southampton, UK
7. Usher Institute of Population Health Sciences and Informatics, University of Edinburgh, Edinburgh, UK
8. British Heart Foundation Centre for Cardiovascular Science, University of Edinburgh, Edinburgh, UK

* The Farr Institute of Health Informatics Research

Correspondence to:

For peer review only

For peer review only - http://bmjopen.bmj.com/site/about/guidelines.xhtml

Simon Sawhney; Institute of Applied Health Sciences; Room 1:040 Polwarth building; Foresterhill;

Aberdeen; AB25 2ZD; simon.sawhney@abdn.ac.uk; +44 (0)1224 437130

For peer review only

For peer review only - http://bmjopen.bmj.com/site/about/guidelines.xhtml

Abstract

Objectives

A rapid growth in the reported rates of acute kidney injury (AKI) has led to calls for greater attention and resources for improving care. However, the reported incidence of acute kidney injury (AKI) also varies more than tenfold between previous studies. Some of this variation is likely to stem from methodological heterogeneity. This study explores the extent of cross-population variation in AKI incidence after minimising heterogeneity.

Design

Population-based cohort study analysing data from electronic health records from three regions in the UK through shared analysis code and harmonised methodology.

Setting

Three populations from Scotland, Wales and England covering three time periods: Grampian 2003, 2007, 2012; Swansea 2007; and Salford 2012.

Participants

All residents in each region, aged 15 years or older.

Main outcome measures

Population incidence of AKI, and AKI phenotype (severity, recovery, recurrence). Determined using shared biochemistry-based AKI episode code and standardised by age and sex.

Results

Respectively, crude AKI rates (per 10,000/year) were: 131, 138, 139, 151 and 124 (p value = 0.095); and after standardisation for age and sex: 147, 151, 146, 146 and 142 (p value = 0.257) for Grampian

For peer review only - http://bmjopen.bmj.com/site/about/guidelines.xhtml

2003, 2007, 2012; Swansea 2007; and Salford 2012. The pattern of variation in crude rates was robust to any modifications of the AKI definition. Across all populations and time periods AKI rates increased substantially with age from ~20 to ~550 per 10,000/year among those aged <40 and ≥70 years.

Conclusion

When harmonised methods are used and age and sex differences are accounted for, a similar high burden of AKI is consistently observed across different populations and time periods (~150 per 10,000/year). There are particularly high rates of AKI among older people. Policy-makers should be careful not draw simplistic assumptions about variation in AKI rates based on comparisons that are not rigorous in methodological terms.

Strengths and limitations

- Previous studies have reported substantial variation in the incidence of AKI between regions and over time, but have involved heterogeneous methods that limit comparability. To our best knowledge, this is the first cross-population study of AKI incidence within one study, with minimised methodological heterogeneity by sharing analysis code across regions.
- By using consistent methods, and real-life, routinely collected health care data, we provide new evidence that the rates of AKI in the UK are similar across different regions and time periods: ~150 events per 10,000/year (1.5% of the population).
- These findings may not be generalisable outside of the regions of the UK in the study. However to enable researchers to replicate this work, we have made publically available our analysis code for identifying and characterising AKI episodes.

For peer review only - http://bmjopen.bmj.com/site/about/guidelines.xhtml

Introduction

The reported outcomes following acute kidney injury (AKI) are consistently poor[1]. Reports of a growth in rates of AKI have led to calls for greater attention and resources for improving care[2], but there is a more than tenfold variation between studies in the reported population incidence of AKI[3-7]. Population based estimates of AKI incidence range from 18 per 10,000/year[3] to 250 per 10,000/year[5] based on changes in serum creatinine over time, and from 3 to 40 per 10,000/year based on hospital episode codes for “non-dialysis requiring AKI”[8,9]. This wide variation is difficult to fully explain[10], but is likely to be due in part to a changing clinical landscape with evolving international AKI criteria[11-14], and different pragmatic interpretations of AKI criteria in research[5,15]. These reasons for variation are all potential sources of bias in clinical studies of AKI (figure 1). Without a clearer understanding of why populations differ, it is challenging (and potentially misleading) to interpret clinical research in context, to make comparisons across populations or over time, or to make informed public health recommendations.

Worldwide, health services are undertaking quality initiatives to increase clinical awareness and improve treatment of AKI[16-19] in order to achieve the International Society of Nephrology (ISN) target of eliminating avoidable deaths from AKI by 2025[20]. To evaluate the effectiveness of these initiatives, it is vital that there is a harmonisation of approaches to clinical research. This means minimising methodological heterogeneity so that the findings of future research are more comparable, and maximising transparency so that trends in disease incidence and outcomes can be understood. Methodological heterogeneity can arise when researchers extract data from different data infrastructures, make different assumptions, and adopt different criteria for identifying events. These steps are particularly important in AKI, because of the recognised challenges of AKI research: it occurs unpredictably, in different clinical locations[21], may be transient[22], and relies on trends rather than absolute values[12-14]. Small differences in how these challenges are handled can alter both the reported incidence and prognosis of AKI[5,15,21,23]. Despite its importance, this information is

For peer review only - http://bmjopen.bmj.com/site/about/guidelines.xhtml

For peer review only

often undocumented or described in insufficient detail for research to be reproduced[24]. We have described these reasons for variation in AKI rates in a conceptual model (figure 1).

Algorithms using blood test data from electronic health records (EHR), offer the potential of an objective common language for observing common diseases in clinical practice, audit and research[25]. In previous work, we developed an extended version of a widely used NHS algorithm for detecting AKI in blood tests[26] which not only flags individual "AKI" blood tests, but also applies phenotyping methods to combine AKI flagged blood tests into clinically meaningful AKI illness episodes grouped by severity, duration, recovery and recurrence[27,28]. Sharing this algorithm between researchers working with different populations provides an opportunity to develop a harmonised approach to clinical research, robustly comparing the burden of AKI across different populations and over time, even when patient-level data cannot be shared. We used this to study of variation in the incidence of AKI across three populations from England, Scotland and Wales. The analysis spans a decade of change in the clinical awareness of AKI[16], change in international AKI criteria[12-14] and change in the emphasis on community surveillance of people with chronic diseases[29-31]. Our aim was to explore the extent of cross-population variation in AKI incidence using real-life data, while minimising heterogeneity through harmonised methods.

Materials and Methods

Population profiles

This study compares datasets created using linked EHR data from primary and secondary care for three UK regions with different "index" years from 2003-2014: Grampian 2003, 2007 and 2012; Abertawe Bro Morgannwg University Health Board (ABMU, referred to in this article as Swansea) 2007; and Salford 2012 (supplemental figure 1). Each dataset involves health data from the UK NHS and includes complete primary and secondary care biochemistry capture for the region. A fourth

For peer review only - http://bmjopen.bmj.com/site/about/guidelines.xhtml

region initially considered for this analysis (from South England) was excluded because initial inspection of the data characteristics revealed that the population capture of the data source was incomplete and might have led to bias in the estimation of AKI. All regions provide public healthcare, free at the point of use.

NHS Grampian, a health authority in Scotland, is served primarily by one large tertiary hospital and another district general hospital. All biochemistry for the dataset was extracted from a single biochemistry department covering the entire regional population[5]. The Grampian dataset was linked with the Scottish Renal Registry to exclude those already receiving chronic renal replacement therapy (RRT), to avoid misclassification of RRT as AKI. Similarly, Salford (North England) represents one borough of Greater Manchester, served by a single NHS hospital and biochemistry laboratory[32]. Read codes (version 2) were used to extract biochemistry information and exclude records from people receiving chronic RRT. In contrast, ABMU (Swansea, Wales) in 2007 covered a region served by four district general hospitals and four laboratories using two information management systems[33,34]. Those receiving chronic RRT could not be directly determined from a register but could be excluded based on the hospital location marked on the blood tests.

To provide further contextual description of these populations we collected information on population mortality and relevant morbidities (renal and vascular) from the Office of National Statistics, UK Renal Registry, and Quality Outcomes Framework (QOF) data entered by GP practices (table 1). Importantly, QOF data represent incentivised recording by GPs of people with a given condition (e.g. chronic kidney disease), rather than actual population prevalences. This means that small differences in prevalence on the disease registers may represent recording practice as well as actual disease prevalence, and should be interpreted with caution.

For peer review only - http://bmjopen.bmj.com/site/about/guidelines.xhtml

Conceptual framework

In figure 1, we provide a conceptual framework for understanding the sources of variation in AKI revealed by our analysis. We sought to minimise "artefactual" methodological differences in AKI episode rates by utilising only datasets where complete data capture (from both hospital and community settings) was possible; by harmonising data preparation and cleaning; and by standardising code sets for identifying AKI episodes. We also accounted for "real" potential sources of variation in AKI rates by performing age and sex standardisation, stratification by baseline eGFR for case-mix differences, and comparing the number of people with blood tests in rapid succession as a surrogate for presence of an acute illness.

Data extraction and processing

This study used a distributed analysis approach to protect the confidentiality of patient-level data. Data were analysed by on-site researchers working from the same code. Non-disclosive summary statistics were aggregated into a single dataset, which was analysed centrally. This ensured that patient-level data were never brought together in a single physical location. All serum creatinine results for each individual were extracted. Creatinine values that were missing, were a non-value (e.g. "sample inadequate", "sample error"), or were lower than the limit for detection of the analyser were excluded. The "Modification of Diet in Renal Disease" (MDRD) study estimated glomerular filtration rate (eGFR) was calculated using the abbreviated 4 variable equation[35]. Finally, to avoid a non-chronological evaluation of samples from different locations, where multiple samples were available for the same individual on a given day, the sample with the highest creatinine value was retained for analysis.

AKI identification and phenotyping

A challenge of AKI clinical research is the operationalisation of precise international AKI criteria in "real-life" data where people do not receive blood tests in a protocolised fashion. Blood tests may

For peer review only - http://bmjopen.bmj.com/site/about/guidelines.xhtml

not have been done at the necessary times to directly observe an acute rise in creatinine from a previous baseline, and assumptions based on available data are required. We identified differences in assumptions for determining AKI as an important potential methodological reason for observed variation in AKI rates (figure 1) and therefore used the exact same definition and analysis code in each region. Kidney Disease Improving Global Outcomes (KDIGO)-based AKI detection and phenotyping algorithm code was applied by separate analysts working locally on each dataset[14]. As summarised in supplemental table 1, these criteria compare each blood test with previous "baseline" results within the last 365 days ("the look-back period") to determine if a recent change has occurred[27]. Where AKI occurred, a "look-forward period" of 90 days was used to follow and phenotype the whole AKI episode. In supplemental figure 2, these look-back and look-forward time periods are illustrated for a single hypothetical patient with respect to a moment of developing AKI within the index year. For those without AKI, the first eGFR of the index year was used as the baseline eGFR. For convenience we used a baseline eGFR <60 ml/min/1.72m^2 as an indicator of chronic kidney disease. Shared Stata code provided the following outputs: number of blood tests consistent with AKI, number of AKI episodes, baseline eGFR, AKI episode severity stage, progression of AKI severity from a lower to higher stage, recovery to baseline within 90 days, and presence of prior AKI episodes in the past three years (i.e. making the episode a recurrent AKI episode).

We also analysed data using more parsimonious versions of the KDIGO criteria: a "narrow interpretation" in which blood tests were only compared if they were no more than a week apart (i.e. restricted to criteria 2 and 3), and a "very narrow interpretation" comparing only tests no more than two days apart (i.e. restricted to criterion 3). If variation was due to a lack of robustness of AKI criteria in the face of estimating baseline from less recent data, these narrower interpretations would be expected to lead to less variation in AKI incidence.

For peer review only - http://bmjopen.bmj.com/site/about/guidelines.xhtml

To ensure uniformity of the application and interpretation of AKI code, a mock dataset of 40 hypothetical patients was developed. This mock dataset deliberately contained unformatted variables and a variety of creatinine trend patterns to represent a full range of data cleaning steps, AKI phenotypes, blood test intervals and interpretation issues. Each analyst used the same code on the test dataset and reproduced the same results before progressing to analysing regional data. We have made the algorithm code, mock dataset, and instructions for their optimal use in Stata freely available from https://github.com/RenalHDRUK.

Statistical analysis

Analyses included the description of baseline characteristics, comparison of both crude and age-sex standardised rates of AKI, and phenotypes of AKI episodes. We also compared AKI rates in subgroups of baseline eGFR (as described above) and individual components of AKI criteria to determine if variations in rate were robust to changes in the AKI definition (table 2). AKI can only be identified when sufficient blood tests have been performed to detect a change. Therefore, to evaluate reasons for residual variation, we described the patterns of blood testing in each region, including the frequency of blood tests, the regularity (e.g. blood tests no more than 2 and 7 days apart) and blood test location (hospital and outpatient/community).

Baseline characteristics included age, sex, the number of people with evidence of renal impairment (eGFR < 60 ml/min/1.73m^2) on their first test in the index year, the number of people with blood tests sufficiently close together for it to be possible to detect an “AKI” result if present (two tests no more than 365 days apart).

We compared population rates of AKI episodes across each region and index year. We compared AKI episode rates using national statistics mid-year population estimates for each region, and then standardised to the England population for 2012[36], a reference population selected as two of the

For peer review only - http://bmjopen.bmj.com/site/about/guidelines.xhtml

three regions provided 2012 data. All AKI episodes in the index year counted towards the overall AKI episode rate. One way ANOVA followed by Tukey's post-hoc test (in the event of significant differences) was used to identify pairwise significant differences in population level AKI episode rates.

For people with sufficient blood tests to potentially detect an episode of AKI (at least two tests no more than 365 days apart), we compared rates within eGFR strata (<30, 30-44, 45-59 and ≥60 ml/min/1.73m^2). The proportion of the population with at least one AKI result based on AKI criteria 1, 2, or 3 (table 2), and the proportion of the population with at least one AKI result based on narrower interpretations of KDIGO criteria (restricting to criteria 2 & 3, or criterion 3 alone) were also recorded. To evaluate the impact of incomplete biochemistry capture, we also recalculated AKI rates using only tests taken from people in hospital. Of note a distinction between hospital inpatient and outpatient results was not possible in Salford.

To evaluate potential sources of residual variation in AKI rates after harmonised analysis we compared patterns of blood testing (number, frequency and location).

Patient involvement

No patients were involved in development of the research question or the design of the study. There are no plans to disseminate the results of the research to study participants.

Results

Populations and baseline characteristics

As described in table 1, populations ranged in size from 193,882 (Salford 2012) to 482,444 people (Grampian 2012) (table 3). Crude reported population mortality rates were higher in Swansea than

For peer review only - http://bmjopen.bmj.com/site/about/guidelines.xhtml

Grampian and Salford, as was the incidence of people starting long term RRT. The recognition of diabetes and cardiovascular diseases in incentivised GP registers was similar across the populations.

Table 2 shows the baseline characteristics of extracted datasets after harmonised data cleaning. The percentage of people with at least two tests no more than 365 days apart varied from 17 – 25% with the fewest in the earliest dataset (Grampian 2003). There was a greater proportion of people tested with renal impairment (estimated glomerular filtration rate, eGFR <60 ml/min/1.73m^2) in 2007 compared to the other years of study.

Incidence of AKI episodes

Table 3 and figure 2 show the differences in crude and standardised rates of AKI episodes for each dataset. A minority of people had more than one AKI episode in the index year. For reporting AKI episode rates (table 3) all episodes are included, whereas for reporting phenotypes of people with an AKI episode, the first episode is described (bottom of table 3 and table 4). Crude AKI rates varied with the lowest in Salford 2012 and highest rate in Swansea 2007 (124-151 per 10,000/year, p value = 0.095). Standardisation by age and sex accounted for residual differences (142-151 per 10,000/year, p value = 0.257), with 95% confidence intervals overlapping in all instances. Age and sex standardised AKI rates varied little between Grampian 2003, 2007 and 2012 (146-151 per 10,000/year). Table 3 also shows that the majority of people developing AKI could be identified using hospital tests alone, and just over half could be identified in each region using a rigid interpretation of KDIGO AKI criteria. Finally, across all populations, the proportion of people developing AKI in the index year increased substantially with increasing age and lower eGFR.

As shown in figure 3, the pattern of variation in crude AKI rates was the same when narrower interpretations of KDIGO AKI criteria were used, comparing only blood tests in the prior 2 and 7

For peer review only - http://bmjopen.bmj.com/site/about/guidelines.xhtml

days. Table 4 shows this pattern was also similar when analysis was limited to each individual component of the AKI criteria, or within strata of baseline eGFR.

AKI phenotypes

Table 4 describes the first AKI episode for people with an AKI episode during the index year. As well as having the highest crude AKI rate, a greater proportion of those with AKI in Swansea were older, had baseline eGFR <60 ml/min/1.73m^2 (37.6%), had a severe AKI episode (15.4% stage 3) had non-recovery at 90 days (45.1%). In Grampian between 2003 and 2012 there was a steady improvement in the proportion of people with renal recovery 90 days after AKI from 42% to 49%.

Further sources of variation

In addition to assessing for age, sex and case-mix differences, we evaluated the blood testing patterns and clinical location contexts of each dataset (figure 4). Figure 4A shows the frequency of blood tests taken grouped by location: hospital inpatient or outpatient/community. Figure 4B shows the proportion of people with blood tests in close succession. In Grampian from 2003 to 2012, community blood testing increased over time but the frequency of hospital inpatient testing remained unchanged. Test location was not available in Salford, but the proportions of people with two blood tests no more than 2 and 7 days apart was lower than in Grampian and Swansea. Figure 1 shows the conceptual framework for understanding these sources of variation.

Discussion

To our knowledge, this is the first multicentre study to systematically evaluate the extent of and reasons for regional and temporal variation in population rates of AKI, using a harmonised methodological approach. There were differences in the crude rates of AKI between datasets, but after accounting for age and sex, standardised rates were strikingly similar (at 140-150 episodes per

For peer review only - http://bmjopen.bmj.com/site/about/guidelines.xhtml

10,000/year, or ~1.5% of the population). The consistently high proportion of people aged over 70 developing AKI was also striking (>5%), and has implications for the planning the future health care requirements of an aging population. This analysis shows the importance of both harmonised methods and standardisation for case-mix prior to any between centre comparisons for description of variation in AKI.

Our analysis provides additional insight into previous reports of a rising AKI incidence in studies based on hospital episode codes or differing AKI definitions[10]. Applying the same KDIGO-based AKI definition to data from the same region, over a ten-year span (2003-2012), the standardised AKI rates in Grampian changed little. Notably, this stability was in spite of an increasing frequency of outpatient/community testing in Grampian (whereas the frequency of hospital inpatient testing changed little over the same period). In addition, our analysis showed similar (albeit reduced) AKI rates across the regions when only hospital blood samples were analysed, or when the AKI definition was limited on only blood tests within the past week. Our analysis also showed a pattern of AKI phenotypes that was consistent with case-mix differences between regions. Swansea, which had the highest all-cause population mortality, also had the highest proportion of AKI phenotypes for severity, AKI progression, and non-recovery.

Between population variation in the prevalence of kidney disease has previously been described for CKD in Ireland[37], Germany[38] and Taiwan[39], as have variation *between* European countries[40]. In our analysis, we have now shown that much of the regional variation in AKI between UK regions can be eliminated by harmonising methods, definitions and correcting for age and sex differences. The stability we report in the AKI incidence over multiple time points in a ten year period is contrary to previous studies from the UK and North America[10]. Given the precautions that we took to minimise heterogeneity, it is possible that some differences reported in previous studies represent a methodological artefact (e.g. data capture or case-mix). Consistent with our findings, a recent study

For peer review only - http://bmjopen.bmj.com/site/about/guidelines.xhtml

of hospital based AKI among people admitted to the Mayo Clinic also found no significant change in AKI rates between 2006-2014 using a consistent creatinine change AKI definition across each year and stratifying by age and sex[41]. Furthermore, in our analysis the pattern of differences in crude AKI rates was robust to modifications of KDIGO criteria using shorter look-back periods.

Our study has caveats common to observational studies, which we have highlighted in a conceptual model that explains the reasons for observed variation in AKI rates (figure 1). In particular, even though we utilised data from three regions with the same social healthcare system (the UK National Health Service), we encountered incomplete population data capture that led to the exclusion of a fourth region from the study. As we note in figure 1, differences in population capture arising out of incomplete data extraction are not necessarily visible to researchers analysing anonymised large datasets. This serves as a critical caution for researchers and policy-makers to avoid making simplistic assumptions that data from different regions are necessarily comparable when they are derived from different sources. We note that while we have used data from GP registers to provide contextual information on the populations, these data need to be interpreted carefully as they also reflect recording practices in primary care rather than solely disease burden. We would also like to remind readers that while we have applied AKI criteria consistently with the same code in each region, where sparse data exist there still may have been bidirectional misclassification between AKI and CKD. Similarly, where AKI has occurred in the context of critical illness, falsely low creatinine values from loss of muscle mass may imply a renal recovery that has not occurred. This is a challenge for all observational studies using routine blood test data. Nevertheless a strength of our analysis is that we have used the same pragmatic approach to this challenge across each of the populations and time periods in the study. Finally, we note that only data from three UK regions were available for inclusion in our study. This is insufficient to describe variation for the whole of the UK and other countries. This article represents a first step towards more harmonised comparisons of AKI across

For peer review only - http://bmjopen.bmj.com/site/about/guidelines.xhtml

populations. We have shared our code with this article (https://github.com/RenalHDRUK) and now invite researchers working with population datasets in other regions to add to our experience.

In conclusion, our analysis shows the need for a robust methodological approach and recognition of case-mix differences when evaluating between-centre and temporal trends in AKI. The sharing of code is key to this approach and we have made our code from this article available for researchers to use. Using this approach we show strikingly similar rates of AKI across different populations from England, Scotland and Wales over a ten year period. A consistently high burden of AKI is apparent with an estimated 1.5% of the UK population experiencing AKI each year, rising to more than 5% per year in the elderly. Current quality initiatives should adopt these methods or similar methods when evaluating the impact of changes in practice on the burden of AKI.

Contributors

CB, SF, SS and SV conceived the study. AM, GD, HR, HH, MJ, SS and TS contributed to collection of the data. AM, CB, HH, HR, JC, NP, PF, PR, SS, SV, TS and SF contributed to analysis of the data. AM, CB, DN, EMH, GD, HH, HR, JC, MJ, NH, NP, PF, PR, RL, SS, SV, TS and SF contributed to interpretation of the data. SS and SF drafted the manuscript with input from AM, CB, DN, EMH, GD, HH, HR, JC, MJ, NH, NP, PF, PR, RL, SV and TS. All authors approved the final version.

Acknowledgements

We would like to acknowledge all the data providers who make anonymised data available for research. This study makes use of anonymised data held in the Secure Anonymised Information Linkage (SAIL) system, which is part of the national e-health records research infrastructure for Wales. We thank the Salford Integrated Record (SIR) board for providing us with the 2014 release of the SIR used in this study.

For peer review only - http://bmjopen.bmj.com/site/about/guidelines.xhtml

Funding

This work was funded by a grant from the UK's Farr Institute for Health Informatics Research (UKHIRN/XFarrRP001). The Farr Institute is supported by a 10-funder consortium: Arthritis Research UK, the British Heart Foundation, Cancer Research UK, the Economic and Social Research Council, the Engineering and Physical Sciences Research Council, the Medical Research Council, the National Institute of Health Research, the National Institute for Social Care and Health Research (Welsh Assembly Government), the Chief Scientist Office (Scottish Government Health Directorates), and the Wellcome (MRC Grant Nos: CIPHER MR/K006525/1, HeRC MR/K006665/1, London MR/K006584/1, Scotland MR/K007017/1). We also acknowledge the data management support of Grampian Data Safe Haven (DaSH) and the associated financial support of NHS Research Scotland, through NHS Grampian investment in the Grampian DaSH. Work on this project was also part funded by the National Institute for Health Research (NIHR) Collaboration for Leadership in Applied Health Research and Care (CLAHRC) Wessex and Health Care and Research Wales. SS is supported by a research training fellowship from the Wellcome Trust to study the outcomes of acute kidney injury (WT102729/Z/13/Z). The funders of this study had no role in study design; collection, analysis, and interpretation of data; writing the report; or the decision to submit the report for publication.

For peer review only

Competing Interests

All authors have completed the ICMJE uniform disclosure form at www.icmje.org/coi_disclosure.pdf and declare: DN reports grants from Informatica, for analyses of the National CKD Audit which was tendered by HQIP (funding from NHS Wales and NHS England), outside the submitted work. SS is supported by a research training fellowship from the Wellcome Trust to study the outcomes of acute kidney injury (WT102729/Z/13/Z). No other support from any organisation for the submitted work; no other financial relationships with any organisations that might have an interest in the submitted

For peer review only - http://bmjopen.bmj.com/site/about/guidelines.xhtml

work in the previous three years; no other relationships or activities that could appear to have influenced the submitted work.

Ethical Approval

Permission for the use of Grampian biochemistry data using routine biochemistry to identify AKI was provided by University of Aberdeen Sponsor, NHS Grampian Caldicott, respective data custodians, NHS Privacy Advisory Committee (ref PAC 33/14), NHS Research and Development Office (project no. 2014RM003) and National Research Ethics Service (reference 14/NW/1371). Permission to analyse SIR data was granted to North West eHealth via the SIR approval board in 2012, which incorporates the appropriate information governance. Further ethical approval was not required, due to the anonymised nature of the data. We thank the SIR board for providing us with the 2014 release of the SIR used in this study. Permissions for using the SAIL databank were gained through application for the SAIL 0505 project, looking at acute kidney injury in Wales. This was reviewed by the Information governance review panel (IGRP) which contains members of the British Medical Association (BMA), National Research Ethics Service (NRES), public health Wales, NHS Wales informatics service (NWIS) and consumer panel.

For peer review only

Data Sharing

No additional data available. Analysis code is freely available from https://github.com/RenalHDRUK.

Transparency declaration

The lead author (SS) affirms that the manuscript is an honest, accurate, and transparent account of the study being reported; that no important aspects of the study have been omitted; and that any discrepancies from the study as planned have been explained.

For peer review only - http://bmjopen.bmj.com/site/about/guidelines.xhtml

References

[1] Chertow GM, Burdick E, Honour M, Bonventre JV, Bates DW. Acute Kidney Injury, Mortality, Length of Stay, and Costs in Hospitalized Patients. Journal of the American Society of Nephrology 2005;16(11):3365-3370.

[2] Hsu RK, Siew ED. The growth of AKI: half empty or half full, it's the size of the glass that matters. Kidney international 2017;92(3):550-553.

[3] Ali T, Khan I, Simpson W, Prescott G, Townend J, Smith W, et al. Incidence and Outcomes in Acute Kidney Injury: A Comprehensive Population-Based Study. Journal of the American Society of Nephrology 2007;18(4):1292-1298.

[4] Hsu C, McCulloch C, Fan D, Ordonez J, Chertow G, Go A. Community-based incidence of acute renal failure. Kidney international 2007;72(2):208-212.

[5] Sawhney S, Fluck N, Marks A, Prescott G, Simpson W, Tomlinson L, et al. Acute kidney injury—how does automated detection perform? Nephrology Dialysis Transplantation 2015;30(11):1853-1861.

[6] Waikar SS, Curhan GC, Wald R, McCarthy EP, Chertow GM. Declining mortality in patients with acute renal failure, 1988 to 2002. Journal of the American Society of Nephrology 2006;17(4):1143-1150.

[7] Bedford M, Stevens P, Wheeler T, Farmer C. What is the real impact of acute kidney injury? BMC Nephrology 2014;15(1):95.

[8] Kolhe NV, Muirhead AW, Wilkes SR, Fluck RJ, Taal MW. The epidemiology of hospitalised acute kidney injury not requiring dialysis in England from 1998 to 2013: retrospective analysis of hospital episode statistics. International journal of clinical practice 2016;70(4):330-339.

[9] Hayward JS, McArthur E, Nash DM, Sontrop JM, Russell SJ, Khan S, et al. Kidney Disease Among Registered Métis Citizens of Ontario: A Population-Based Cohort Study. Canadian Journal of Kidney Health and Disease 2017;4:2054358117703071.

[10] Siew ED, Davenport A. The growth of acute kidney injury: a rising tide or just closer attention to detail? Kidney international 2015;87(1):46-61.

[11] Van Biesen W, Vanholder R, Lameire N. Defining Acute Renal Failure: RIFLE and Beyond. Clinical Journal of the American Society of Nephrology 2006;1(6):1314-1319.

[12] Bellomo R, Ronco C, Kellum J, Mehta R, Palevsky P, the Aw. Acute renal failure - definition, outcome measures, animal models, fluid therapy and information technology needs: the Second International Consensus Conference of the Acute Dialysis Quality Initiative (ADQI) Group. Critical Care 2004;8(4):R204-R212.

[13] Mehta RL, Kellum JA, Shah SV, Molitoris BA, Ronco C, Warnock DG, et al. Acute Kidney Injury Network: report of an initiative to improve outcomes in acute kidney injury. Critical care 2007;11(2):R31.

For peer review only - http://bmjopen.bmj.com/site/about/guidelines.xhtml

[14] Kidney Disease: Improving Global Outcomes (KDIGO) Acute Kidney Injury Work Group. KDIGO Clinical Practice Guideline for Acute Kidney Injury. Kidney international 2012;2:1-138.

[15] Lafrance JP, Miller DR. Defining acute kidney injury in database studies: the effects of varying the baseline kidney function assessment period and considering CKD status. American Journal of Kidney Diseases 2010;56(4):651-660.

[16] Stewart J, Findlay G, Smith N, Kelly K, Mason M. Adding Insult to Injury: A review of the care of patients who died in hospital with a primary diagnosis of acute kidney injury. London: National Confidential Inquiry into Patient Outcome and Death.; 2009.

[17] Silver SA, Adu D, Agarwal S, Gupta KL, Lewington AJP, Pannu N, et al. Strategies to Enhance Rehabilitation After Acute Kidney Injury in the Developing World. Kidney International Reports 2017;2(4):579-593.

[18] Think Kidneys. Think Kidneys. 2015; Available at: https://www.thinkkidneys.nhs.uk/. Accessed 07/04, 2017.

[19] Mehta R, Bagga A, Patibandla R, Chakravarthi R. Detection and Management of AKI in the Developing World: The 18th Acute Disease Quality Initiative (ADQI) International Consensus Conference. Kidney International Reports 2017;2(4):515-518.

[20] Mehta RL, Cerda J, Burdmann EA, Tonelli M, Garcia-Garcia G, Jha V, et al. International Society of Nephrology's 0by25 initiative for acute kidney injury (zero preventable deaths by 2025): a human rights case for nephrology. Lancet 2015;385(9987):2616-2643.

[21] Sawhney S, Fluck N, Fraser SD, Marks A, Prescott GJ, Roderick PJ, et al. KDIGO-based acute kidney injury criteria operate differently in hospitals and the community—findings from a large population cohort. Nephrology Dialysis Transplantation 2016;31(6):922-929.

[22] Uchino S, Bellomo R, Bagshaw SM, Goldsmith D. Transient azotaemia is associated with a high risk of death in hospitalized patients. Nephrology Dialysis Transplantation 2010;25(6):1833-1839.

[23] Sawhney S, Mitchell M, Marks A, Fluck N, Black C. Long-term prognosis after acute kidney injury (AKI): what is the role of baseline kidney function and recovery? A systematic review. BMJ Open 2015;5(1):e006497.

[24] Playford CJ, Gayle V, Connelly R, Gray AJG. Administrative social science data: The challenge of reproducible research. Big Data & Society 2016;3(2):2053951716684143.

[25] Bagshaw SM, Goldstein SL, Ronco C, Kellum JA. Acute kidney injury in the era of big data: the 15th Consensus Conference of the Acute Dialysis Quality Initiative (ADQI). Canadian Journal of Kidney Health and Disease 2016;3(1):1-5.

[26] NHS England. Patient safety alert on standardising the early identification of Acute Kidney Injury. 2014; Available at: http://www.england.nhs.uk/2014/06/09/psa-aki/. Accessed 07/04, 2017.

[27] Sawhney S, Marks A, Fluck N, Levin A, Prescott GJ, Black C. Intermediate and Long-Term Outcomes of Survivors of Acute Kidney Injury Episodes: A Large Population-Based Cohort Study. American Journal of Kidney Diseases 2017;69(1):18-28.

For peer review only - http://bmjopen.bmj.com/site/about/guidelines.xhtml

[28] Sawhney S, Marks A, Fluck N, Levin A, McLernon D, Prescott G, et al. Post-discharge kidney function is associated with subsequent ten-year renal progression risk among survivors of acute kidney injury. Kidney international 2017;92(2):440-452.

[29] Kidney Disease: Improving Global Outcomes (KDIGO) CKD Work Group. KDIGO Clinical Practice Guideline for the Evaluation and Management of Chronic Kidney Disease. Kidney international 2013;3:1-150.

[30] Yancy CW, Jessup M, Bozkurt B, Butler J, Casey DE, Drazner MH, et al. 2013 ACCF/AHA Guideline for the Management of Heart Failure. Circulation 2013;128(16):e240.

[31] Guideline Development Group, Bilo H, Coentrao L, Couchoud C, Covic A, De Sutter J, et al. Clinical Practice Guideline on management of patients with diabetes and chronic kidney disease stage 3b or higher (eGFR <45 mL/min). Nephrology Dialysis Transplantation 2015;30:ii1-ii142.

[32] Fraccaro P, van der Veer SN, Brown B, Prosperi M, O'Donoghue D, Collins GS, et al. An external validation of models to predict the onset of chronic kidney disease using population-based electronic health records from Salford, UK. BMC Medicine 2016;14(1):104.

[33] Ford DV, Jones KH, Verplancke J, Lyons RA, John G, Brown G, et al. The SAIL Databank: building a national architecture for e-health research and evaluation. BMC Health Services Research 2009;9(1):157.

[34] Lyons RA, Jones KH, John G, Brooks CJ, Verplancke J, Ford DV, et al. The SAIL databank: linking multiple health and social care datasets. BMC Medical Informatics and Decision Making 2009;9(1):3.

[35] Levey AS, Stevens LA, Schmid CH, Zhang Y, Castro AF, Feldman HI, et al. A New Equation to Estimate Glomerular Filtration Rate. Annals of Internal Medicine 2009;150(9):604-612.

[36] Roalfe AK, Holder RL, Wilson S. Standardisation of rates using logistic regression: a comparison with the direct method. BMC Health Services Research 2008;8(1):275.

[37] Stack AG, Casserly LF, Cronin CJ, Chernenko T, Cullen W, Hannigan A, et al. Prevalence and variation of Chronic Kidney Disease in the Irish health system: initial findings from the National Kidney Disease Surveillance Programme. BMC Nephrology 2014;15(1):185.

[38] Aumann N, Baumeister SE, Rettig R, Lieb W, Werner A, Doring A, et al. Regional Variation of Chronic Kidney Disease in Germany: Results From Two Population-Based Surveys. Kidney and Blood Pressure Research 2015;40(3):231-243.

[39] Yap Y, Chuang K, Chiang C, Chuang H, Lu S. Geographic Variation of Chronic Kidney Disease Prevalence: Correlation with the Incidence of Renal Cell Carcinoma or Urothelial Carcinoma? BioMed Research International 2015;2015:427084.

[40] Brück K, Stel VS, Gambaro G, Hallan S, Völzke H, Ärnlöv J, et al. CKD Prevalence Varies across the European General Population. Journal of the American Society of Nephrology 2016;27(7):2135-2147.

[41] Kashani K, Shao M, Li G, Williams AW, Rule AD, Kremers WK, et al. No increase in the incidence of acute kidney injury in a population-based annual temporal trends epidemiology study. Kidney international 2017;(in press).

For peer review only

For peer review only - http://bmjopen.bmj.com/site/about/guidelines.xhtml

For peer review only

For peer review only - http://bmjopen.bmj.com/site/about/guidelines.xhtml

Tables and Figures

Table 1 – Contextual information on the populations in this study

	Grampian 2003	Grampian 2007	Grampian 2012	Swansea 2007	Salford 2012
**Index year for assessing incident AKI episodes**	2003	2007	2012	2007	2012
**Mid-year regional population (all ages) during index year[1]**	529,360	548,290	573,400	499,400	237,085
**Mid-year regional adult population (age ≥ 15 years) during index year[1]**	438,332	458,900	482,444	415,500	193,882
**Percentage of population in urban settlements of > 10,000 people[2]**	49.3%	51.8%	52.1%	81.7%	99.9%
**Regional crude all-cause mortality rate ages 15+ (index year/100,000)[1]**	1192	1154	1093	1334	1135
**Crude adult incidence of chronic RRT per million population (UKRR)[3]**	98	102	93	167	85
**Prevalence of chronic kidney disease per 100 people (QOF)[4]**	n/a[5]	2.6	3.3	1.8	3.0
**Prevalence of coronary heart disease per 100 people (QOF)[4]**	4.1	4.0	3.9	4.1	3.9
**Prevalence of diabetes registration per 100 people (QOF)[4]**	3.0	3.3	4.2	4.3	4.5
**Prevalence of heart failure registration per 100 people (QOF)[4]**	n/a[4]	0.8	0.8	1.0	0.9
**Prevalence of hypertension registration per 100 people (QOF)[4]**	11.0	11.7	13.2	12.5	13.8
**Prevalence of stroke & TIA registration per 100 people (QOF)[4]**	1.6	1.7	1.9	2.1	1.8
**Number of biochemistry departments for whole region**	One department covers in and outpatient, community and private tests	One department covers in and outpatient, community and private tests	One department covers in and outpatient, community and private tests	Four departments cover in and outpatients, community and private tests	One department covers in and outpatient and community tests. Privately obtained samples unavailable
**Means of excluding samples belonging to people on long term RRT from dataset**	Link to Scottish Renal Registry	Link to Scottish Renal Registry	Link to Scottish Renal Registry	Removing samples from locations where renal replacement is performed, including intensive care unit	Read code screening
**IDMS aligned creatinine assay**	Yes	Yes	Yes	From 2007	Yes

[1] From the Office of National Statistics
[2] From the 2011 National Census in England and Wales and Scottish Government Urban Rural Classification
[3] From the UK Renal Registry (UKRR) annual reports
[4] Quality Outcomes Framework (QOF) data is incentivised information entered by GP practices. Not recorded in Grampian in 2003, for which 2004 data is provided where available.
[5] Data not available

For peer review only - http://bmjopen.bmj.com/site/about/guidelines.xhtml

Table 2 – Baseline characteristics for each dataset

	Grampian 2003		Grampian 2007		Grampian 2012		Swansea 2007		Salford 2012	
	Patient total	(%)[1]	Patient total	(%)	Patient total	(%)	Patient total	(%)	Patient total	(%)
Adult resident population (aged ≥ 15)	438332		458900		482444		415500		193882	
**Population ascertainment of renal impairment (eGFR < 60 ml/min/1.73m^2) in index year**										
No tests during index year	311922	(71.2)	303673	(66.2)	301992	(62.6)	253531	(61.0)	116977	(60.3)
eGFR ≥60 [2]	101595	(23.2)	120854	(26.3)	158736	(32.9)	129959	(31.3)	66890	(34.5)
eGFR <60 [2]	24805	(5.7)	34373	(11.3)	21716	(4.5)	32010	(7.7)	10015	(5.2)
**Sufficiency of tests to enable AKI detection**										
People with no tests during index year	311922	(71.2)	303673	(66.2)	301992	(62.6)	253531	(61.0)	116977	(60.3)
People with insufficient tests	52602	(12.0)	57788	(12.6)	69239	(14.4)	59839	(14.4)	31467	(16.2)
People with ≥2 tests within 365 days	73808	(16.8)	97439	(21.2)	111213	(23.1)	102130	(24.6)	45438	(23.4)
**Characteristics of people with ≥ 2 tests within 365 days**										
Proportion female	40413	(54.8)	53061	(54.5)	60330	(54.2)	55685	(54.5)	24723	(54.4)
Median age (IQR)	63	(48-74)	63	(50-75)	63	(49-74)	64	(51-75)	63	(49-74)
eGFR <60 [2]	18573	(25.2)[3]	28274	(29.0)	18679	(20.2)	25952	(25.4)	8541	(18.8)

[1] Expressed as a percentage of total residents unless specified otherwise
[2] First estimated glomerular filtration rate in index year (ml/min/1.73m^2)
[3] Expressed as a percentage of people with ≥ 2 tests within 365 days

For peer review only

For peer review only - http://bmjopen.bmj.com/site/about/guidelines.xhtml

Table 3 – Crude and standardised rates of AKI episodes, and components of AKI criteria

	Grampian 2003 (Rate per 10,000)[1]	Grampian 2007 (Rate per 10,000)	Grampian 2012 (Rate per 10,000)	Swansea 2007 (Rate per 10,000)	Salford 2012 (Rate per 10,000)
Adult resident population	438332	458900	482444	415500	193882
**AKI incidence rates**					
Crude AKI incidence (95% CI)	131.2 (127.7-134.7)	138.3 (134.9-141.7)	139.1 (135.8-142.4)	151.1 (147.4-154.8)	124.3 (118.8-129.8)
Age-sex standardised AKI incidence (95% CI)	147.2 (143.3-151.1)	150.6 (146.9-154.3)	146.3 (142.8-149.8)	145.6 (142.0-149.2)	141.8 (136.2-147.4)
Total AKI episodes	5749 (131)	6346 (138)	6711 (139)	6266 (151)	2399 (124)
People with AKI	5362 (122)	5930 (129)	6277 (130)	5847 (141)	2208 (114)
**Subgroups of people with AKI**					
AKI using hospital tests only	4386 (100)	4739 (103)	4492 (93)	4432 (107)	n/a[2]
Rigid KDIGO criteria	3436 (78)	3803 (83)	3617 (75)	3469 (83)	1114 (57)
People meeting 2d criterion	2486 (57)	2831 (62)	2714 (56)	2424 (58)	741 (38)
People meeting 7d criterion	2488 (57)	2698 (56)	2664 (55)	2611 (63)	821 (42)
People meeting 8-90d criterion	2619 (60)	2830 (59)	3351 (69)	3287 (79)	1163 (60)
People meeting 91-365d criterion	1408 (32)	1528 (32)	1850 (38)	1591 (38)	737 (38)
**People with AKI in age strata**					
≥70 years	3205 (562)	3561 (587)	3705 (572)	3785 (584)	1299 (544)
40-69 years	1765 (88)	1903 (89)	2021 (89)	1699 (89)	740 (92)
<40 years	392 (22)	466 (25)	551 (29)	363 (23)	169 (19)
**People with AKI in eGFR strata among people with at least two tests within 365 days (rates expressed within strata of tested individuals at risk)**					
Baseline eGFR ≥60	3612 (654)	3874 (560)	4419 (478)	3648 (479)	1512 (410)
Baseline eGFR 45-59	809 (673)	940 (496)	894 (756)	1044 (618)	323 (607)
Baseline eGFR 30-44	597 (1222)	723 (1000)	661 (1282)	732 (1097)	202 (867)
Baseline eGFR <30	344 (2064)	393 (1861)	303 (1781)	423 (1778)	171 (1921)

[1]Rate expressed per 10,000 residents unless specified otherwise
[2]Location data not available

For peer review only

Table 4 – Phenotype of AKI episodes

	Grampian 2003		Grampian 2007		Grampian 2012		Swansea 2007		Salford 2012	
	Total people	(%)	Total people	(%)	Total people	(%)	Total people	(%)	Total people	(%)
**People with AKI**	5362		5930		6277		5847		2208	
Proportion female	2899	(54.1)	3256	(54.9)	3443	(54.9)	3195	(54.6)	1250	(56.6)
Median age (IQR)	73	(61-81)	74	(61-82)	74	(60-82)	76	(64-84)	74	(61-83)
**Peak AKI severity stage for first episode**										
stage 1	3720	(69.4)	4211	(71.0)	4389	(69.9)	3720	(63.6)	1435	(65.0)
stage 2	1014	(18.9)	1063	(17.9)	1174	(18.7)	1224	(20.9)	451	(20.4)
stage 3	628	(11.7)	656	(11.1)	714	(11.4)	903	(15.4)	322	(14.6)
AKI stage progression	817	(15.2)	792	(13.4)	850	(13.5)	900	(15.4)	300	(13.6)
**Baseline eGFR for first episode (ml/min/1.73m^2)**										
≥60	3612	(67.4)	3874	(65.3)	4419	(70.4)	3648	(62.4)	1512	(68.5)
45-59	809	(15.1)	940	(15.9)	894	(14.2)	1044	(17.9)	323	(14.6)
30-44	597	(11.1)	723	(12.2)	661	(10.5)	732	(12.5)	202	(9.1)
<30	344	(6.4)	393	(6.6)	303	(4.8)	423	(7.2)	171	(7.7)
**Prior AKI episodes detected in last 3 years**										
No prior episodes	4415	(82.3)	4847	(81.7)	5052	(80.5)	4824	(82.5)	1708	(77.4)
1 prior episode	723	(13.5)	833	(14.0)	897	(14.3)	784	(13.4)	349	(15.8)
2 or more prior episodes	224	(4.2)	250	(4.2)	328	(5.2)	239	(4.1)	151	(6.8)
Prior AKI within 1 year	414	(7.7)	459	(7.7)	492	(7.8)	488	(8.3)	216	(9.8)
**Renal recovery to within 20% of baseline**										
Renal recovery	2239	(41.8)	2588	(43.6)	3077	(49.0)	2156	(36.9)	970	(43.9)
Renal non-recovery	2203	(41.1)	2387	(40.3)	2245	(35.8)	2635	(45.1)	820	(37.1)
Repeat samples not available	920	(17.2)	955	(16.1)	955	(15.2)	1056	(18.1)	418	(18.9)

[1]Expressed as a percentage of people with at least one AKI episode
[2]Insufficient biochemistry data available to report on the previous 3 years

For peer review only

Figure 1 – Conceptual framework for the reasons for cross-population differences in AKI rates

Figure 2 – Crude and age-sex standardised rate of AKI episodes

Figure 3 – Crude AKI rates using different interpretations of the KDIGO-based AKI definition

Figure 4 – Patterns of blood testing by clinical location (4A), and by test regularity (4B)

For peer review only

For peer review only - http://bmjopen.bmj.com/site/about/guidelines.xhtml

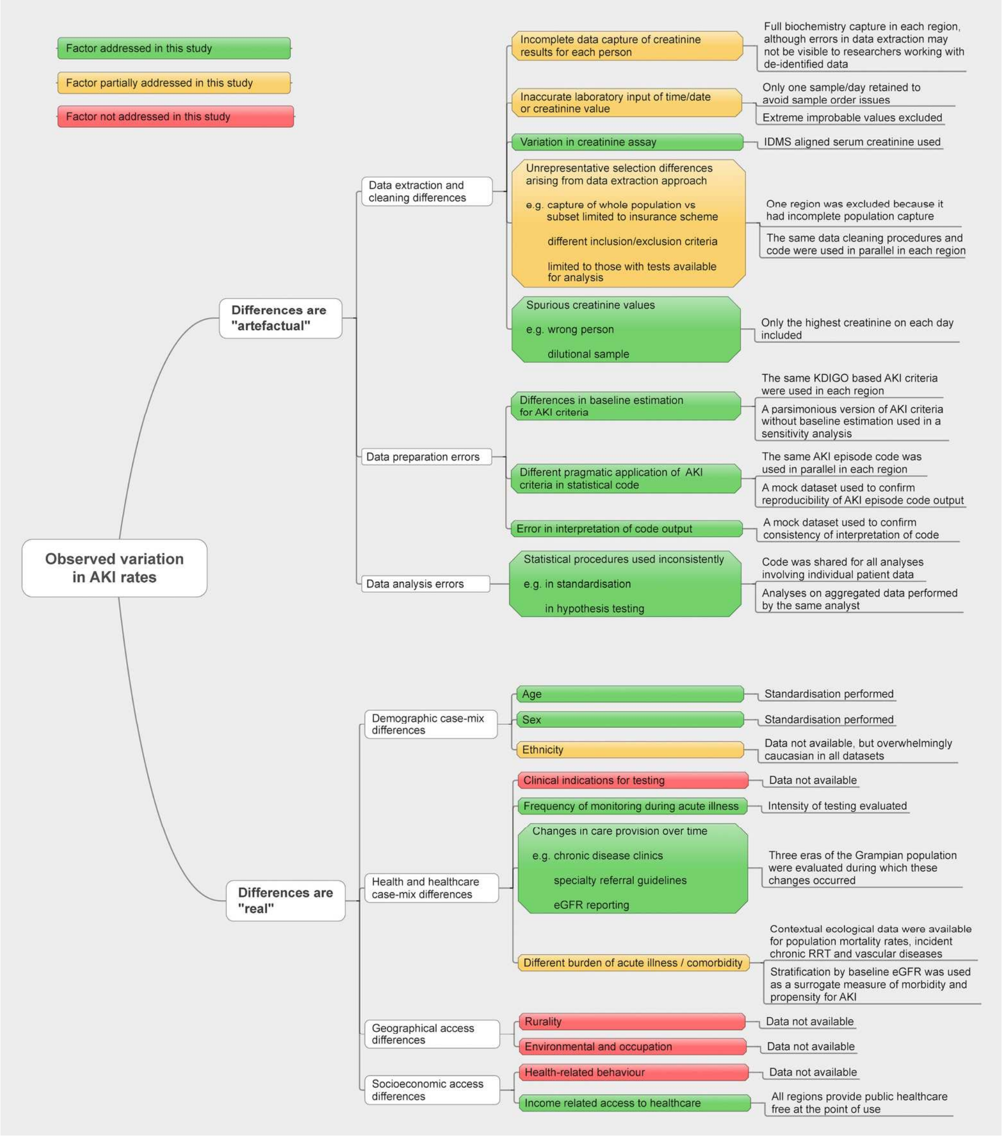

Conceptual framework for the reasons for cross-population differences in AKI rates

181x205mm (300 x 300 DPI)

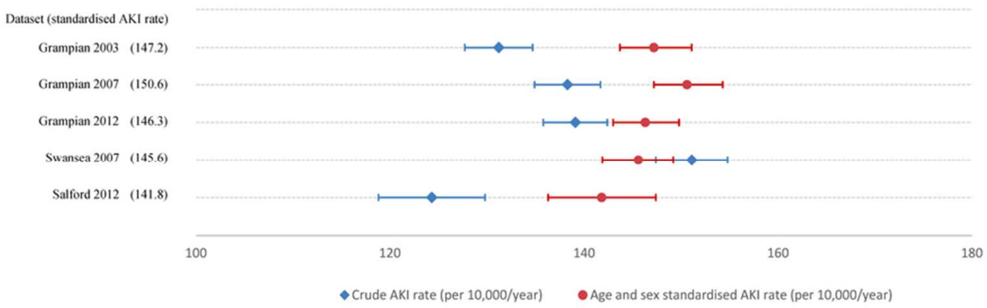

Crude and age-sex standardised rate of AKI episodes

75x25mm (300 x 300 DPI)

peer review only

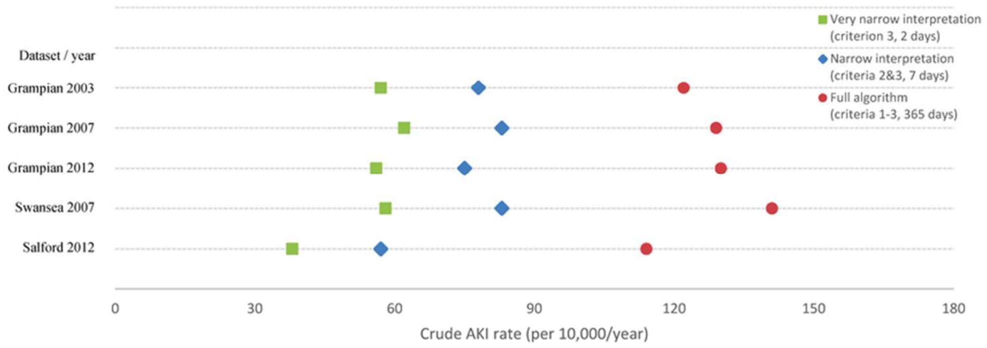

Crude AKI rates using different interpretations of the KDIGO-based AKI definition

73x26mm (300 x 300 DPI)

For peer review only

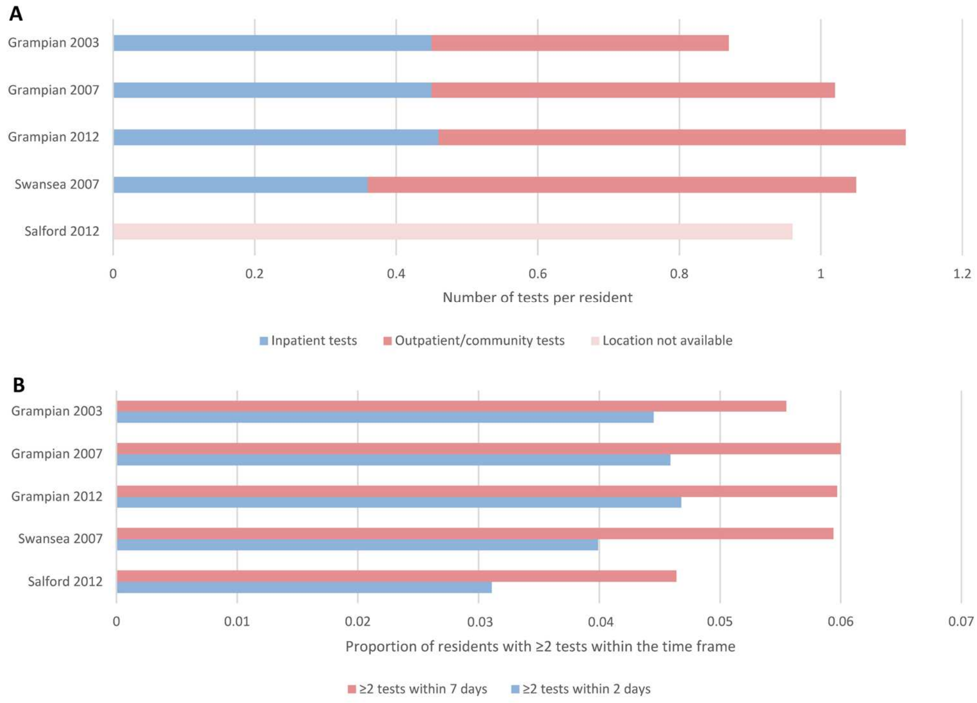

Patterns of blood testing by clinical location (4A), and by test regularity (4B)

155x111mm (300 x 300 DPI)

Supplemental table 1 – AKI definition and phenotype criteria for this study

**AKI Criteria**	**AKI definition**
Criterion 1	Serum creatinine ≥1.5 times higher than the median of all creatinine values 8-90 days ago, or 91-365 days ago if no tests between 8-90 days
Criterion 2	Serum creatinine ≥1.5 times higher than the lowest creatinine within 7 days
Criterion 3	Serum creatinine >26 μmol/L higher than the lowest creatinine within 48 hours
**AKI severity**	**Staging definition (based on peak creatinine within 90 days of diagnosis)**
Stage 1	Rise in creatinine of >26 μmol/L; or index/baseline ratio ≥1.5 and <2
Stage 2	Index/baseline ratio ≥2 and <3
Stage 3	Index/baseline ratio ≥3; or ≥1.5 and index creatinine >354 μmol/L
**Prior AKI episodes**	**Prior AKI definition**
No prior AKI	AKI episode not preceded by any previous AKI episodes in the prior 3 years
Prior AKI	AKI episode preceded by at least one previous AKI episode in the prior 3 years
Recent prior AKI	AKI episode preceded by at least one previous AKI episode in the prior 1 year
**90 day AKI recovery**	**Recovery definition**
Recovery	Last creatinine within 90 days of AKI <1.2 times higher than the baseline creatinine at diagnosis
Non-recovery	Last creatinine within 90 days of AKI ≥1.2 times higher than the baseline creatinine at diagnosis, or still receiving acute RRT
"Untested"	No repeat blood tests taken within 90 days of AKI diagnosis

Abbreviations: AKI, acute kidney injury; RRT, renal replacement therapy

For peer review only

Supplementary figure 1 – Map of the UK populations in this analysis

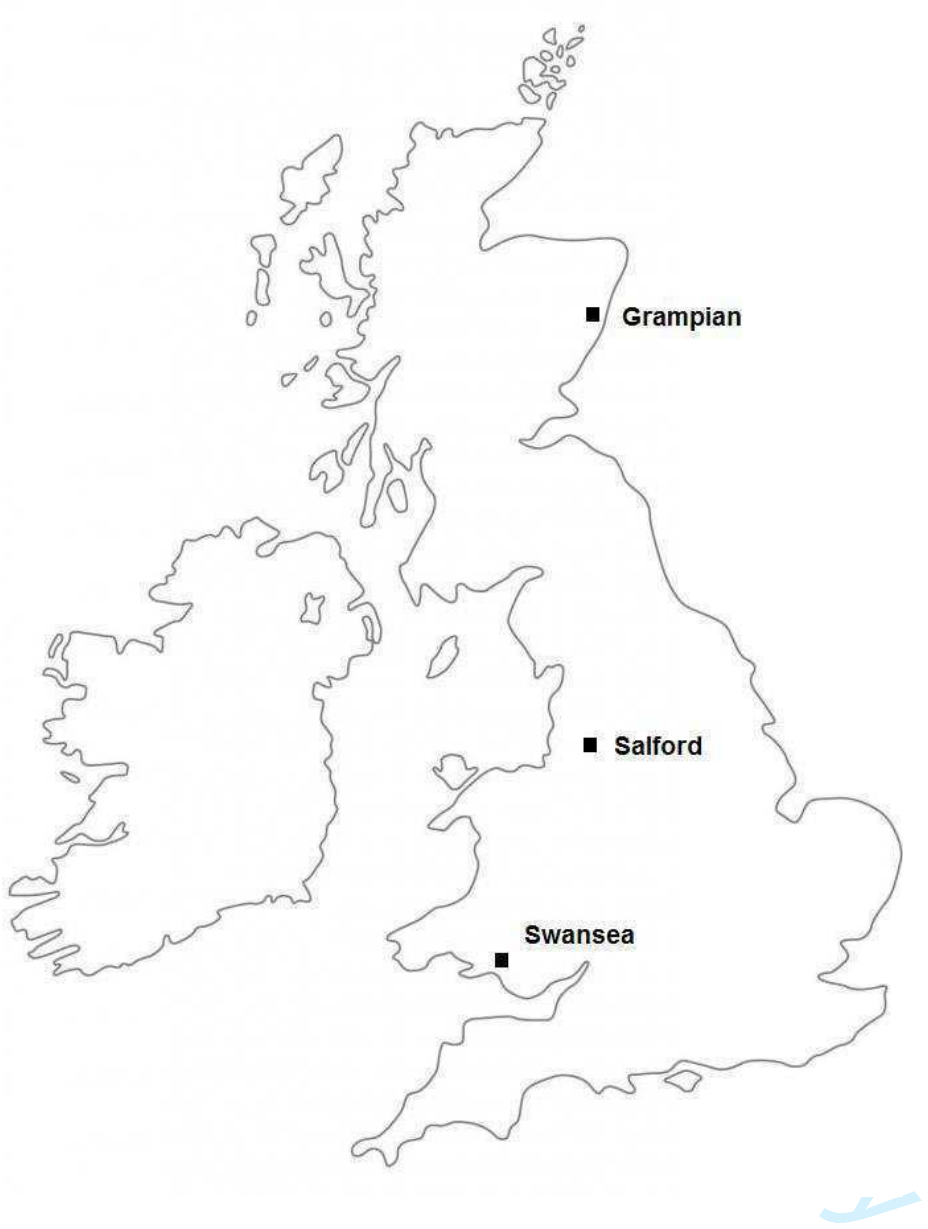

Supplemental figure 2 – Hypothetical patient illustrating look-back (for baseline) and look-forward (for AKI episode phenotyping) time periods from the start of an AKI episode

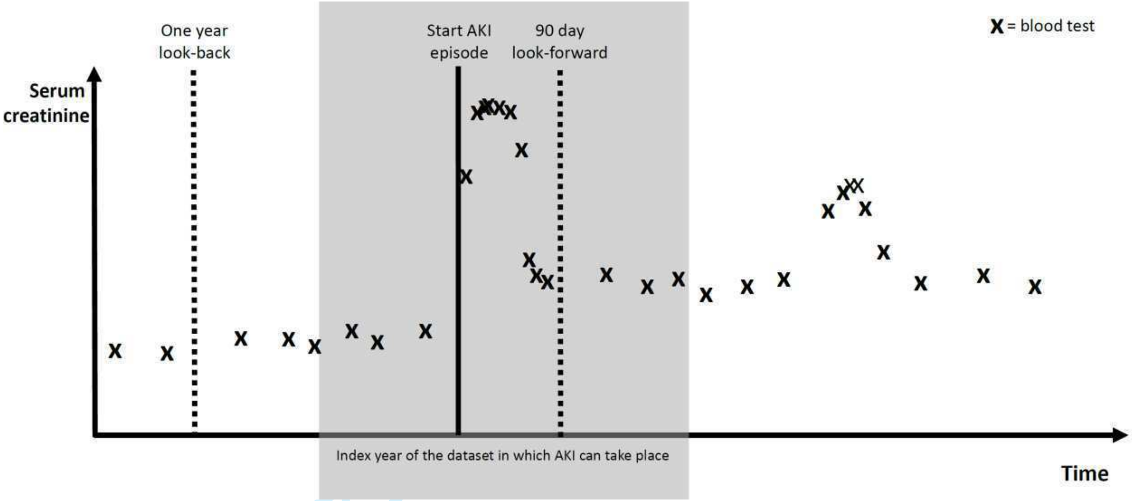

For peer review only - http://bmjopen.bmj.com/site/about/guidelines.xhtml

STROBE Statement—Checklist of items that should be included in reports of ***cohort studies***

	Item No	Recommendation	Pages
**Title and abstract**	1	(*a*) Indicate the study's design with a commonly used term in the title or the abstract	3
		(*b*) Provide in the abstract an informative and balanced summary of what was done and what was found	3
**Introduction**			
Background/rationale	2	Explain the scientific background and rationale for the investigation being reported	5
Objectives	3	State specific objectives, including any prespecified hypotheses	6
**Methods**			
Study design	4	Present key elements of study design early in the paper	6
Setting	5	Describe the setting, locations, and relevant dates, including periods of recruitment, exposure, follow-up, and data collection	6
Participants	6	(*a*) Give the eligibility criteria, and the sources and methods of selection of participants. Describe methods of follow-up	7, 8
		(*b*) For matched studies, give matching criteria and number of exposed and unexposed	n/a
Variables	7	Clearly define all outcomes, exposures, predictors, potential confounders, and effect modifiers. Give diagnostic criteria, if applicable	9, 10
Data sources/ measurement	8*	For each variable of interest, give sources of data and details of methods of assessment (measurement). Describe comparability of assessment methods if there is more than one group	8-10
Bias	9	Describe any efforts to address potential sources of bias	9
Study size	10	Explain how the study size was arrived at	6, 11, table 1
Quantitative variables	11	Explain how quantitative variables were handled in the analyses. If applicable, describe which groupings were chosen and why	10, 11
Statistical methods	12	(*a*) Describe all statistical methods, including those used to control for confounding	10,11
		(*b*) Describe any methods used to examine subgroups and interactions	10,11
		(*c*) Explain how missing data were addressed	n/a
		(*d*) If applicable, explain how loss to follow-up was addressed	n/a
		(*e*) Describe any sensitivity analyses	11
**Results**			
Participants	13*	(a) Report numbers of individuals at each stage of study—eg numbers potentially eligible, examined for eligibility, confirmed eligible, included in the study, completing follow-up, and analysed	11, tables 1 & 2
		(b) Give reasons for non-participation at each stage	7
		(c) Consider use of a flow diagram	n/a
Descriptive data	14*	(a) Give characteristics of study participants (eg demographic, clinical, social) and information on exposures and potential confounders	Tables 1 and 2
		(b) Indicate number of participants with missing data for each variable of interest	n/a
		(c) Summarise follow-up time (eg, average and total amount)	n/a
Outcome data	15*	Report numbers of outcome events or summary measures over time	12, 13, table 3

For peer review only - http://bmjopen.bmj.com/site/about/guidelines.xhtml
For peer review only

Main results	16	(*a*) Give unadjusted estimates and, if applicable, confounder-adjusted estimates and their precision (eg, 95% confidence interval). Make clear which confounders were adjusted for and why they were included	12, 13, table 3
		(*b*) Report category boundaries when continuous variables were categorized	Table 3
		(*c*) If relevant, consider translating estimates of relative risk into absolute risk for a meaningful time period	n/a
Other analyses	17	Report other analyses done—eg analyses of subgroups and interactions, and sensitivity analyses	Tables 3 & 4
**Discussion**			
Key results	18	Summarise key results with reference to study objectives	13, 14
Limitations	19	Discuss limitations of the study, taking into account sources of potential bias or imprecision. Discuss both direction and magnitude of any potential bias	15
Interpretation	20	Give a cautious overall interpretation of results considering objectives, limitations, multiplicity of analyses, results from similar studies, and other relevant evidence	16
Generalisability	21	Discuss the generalisability (external validity) of the study results	15
**Other information**			
Funding	22	Give the source of funding and the role of the funders for the present study and, if applicable, for the original study on which the present article is based	17

*Give information separately for exposed and unexposed groups.

Note: An Explanation and Elaboration article discusses each checklist item and gives methodological background and published examples of transparent reporting. The STROBE checklist is best used in conjunction with this article (freely available on the Web sites of PLoS Medicine at http://www.plosmedicine.org/, Annals of Internal Medicine at http://www.annals.org/, and Epidemiology at http://www.epidem.com/). Information on the STROBE Initiative is available at http://www.strobe-statement.org.

For peer review only

For peer review only - http://bmjopen.bmj.com/site/about/guidelines.xhtml
